# Inhibition of IRAK4 by microbial trimethylamine blunts metabolic inflammation and ameliorates glycemic control

**DOI:** 10.1038/s42255-025-01413-8

**Published:** 2025-12-08

**Authors:** Julien Chilloux, Francois Brial, Amandine Everard, David Smyth, Petros Andrikopoulos, Liyong Zhang, Hubert Plovier, Antonis Myridakis, Lesley Hoyles, José Maria Moreno-Navarrete, Jèssica Latorre Luque, Viviana Casagrande, Rossella Menghini, Blerina Ahmetaj-Shala, Christine Blancher, Laura Martinez-Gili, Selin Gencer, Jane F. Fearnside, Richard H. Barton, Ana Luisa Neves, Alice R. Rothwell, Christelle Gérard, Sophie Calderari, Mark J. Williamson, Julian E. Fuchs, Lata Govada, Claire L. Boulangé, Saroor Patel, James Scott, Mark Thursz, Naomi Chayen, Robert C. Glen, Nigel J. Gooderham, Jeremy K. Nicholson, Massimo Federici, José Manuel Fernández-Real, Dominique Gauguier, Peter P. Liu, Patrice D. Cani, Marc-Emmanuel Dumas

**Affiliations:** 1 Section of Biomolecular Medicine, Division of Systems Medicine, Department of Metabolism, Digestion and Reproduction, Imperial College London, London, UK; 2 University Paris Cité, INSERM U1132, Biologie de l’os et du cartilage (BIOSCAR), Paris, France; 3 Université catholique de Louvain, Louvain Drug Research Institute, Metabolism and Nutrition Research Group, Brussels, Belgium; 4 Walloon Excellence in Life Sciences and Biotechnology (WELBIO) Department, WEL Research Institute, Wavre, Belgium; 5 University of Ottawa Heart Institute, Ottawa, Ontario Canada; 6 Department of Biosciences, Nottingham Trent University, Clifton Campus, Nottingham, UK; 7 Department of Endocrinology, Diabetes and Nutrition, University Hospital of Girona ‘Dr Josep Trueta’, Girona, Spain; 8 Institut d’Investigació Biomèdica de Girona (IdibGi), Girona, Spain; 9 CIBER Fisiopatología de la Obesidad y Nutrición (CIBERobn), Instituto de Salud Carlos III, Madrid, Spain; 10 Department of Systems Medicine, University of Rome Tor Vergata, Via Montpellier, Rome, Italy; 11 Section of Cardio-Respiratory Interface, National Heart & Lung Institute, Imperial College London, London, UK; 12 Wellcome Trust Centre for Human Genetics, Roosevelt Drive, University of Oxford, Oxford, UK; 13 University Paris Cité, Functional and Adaptive Biology, CNRS UMR 8251, Paris, France; 14 University Paris-Saclay, INRAE, Jouy-en-Josas, France; 15 Centre for Molecular Informatics, Department of Chemistry, University of Cambridge, Cambridge, UK; 16 Section of Hepatology & Gastroenterology, Division of Digestive Diseases, Department of Metabolism, Digestion and Reproduction, Imperial College London, London, UK; 17 The Australian National Phenome Centre, Health Futures Institute, Murdoch University, Harry Perkins Institute, Perth, Australia; 18 Department of Medical Sciences, School of Medicine, University of Girona, Girona, Spain; 19 Victor Phillip Dahdaleh Institute of Genomic Medicine at McGill University, Montreal, Quebec Canada; 20 Institute of Experimental and Clinical Research (IREC), UCLouvain, Université catholique de Louvain, Brussels, Belgium; 21 European Genomic Institute for Diabetes, U1283 INSERM, UMR8199 CNRS, Institut Pasteur de Lille, Lille University Hospital, University of Lille, Lille, France

**Keywords:** Kinases, Type 2 diabetes, Microbiome, Metabolomics, Metabolism

## Abstract

The global type 2 diabetes epidemic is a major health crisis. Although the microbiome has roles in the onset of insulin resistance (IR), low-grade inflammation and diabetes, the microbial compounds controlling these processes remain to be discovered. Here, we show that the microbial metabolite trimethylamine (TMA) decouples inflammation and IR from diet-induced obesity by inhibiting interleukin-1 receptor-associated kinase 4 (IRAK4), a central kinase in the Toll-like receptor pathway sensing danger signals. TMA blunts TLR4 signalling in primary human hepatocytes and peripheral blood monocytic cells and rescues mouse survival after lipopolysaccharide-induced septic shock. Genetic deletion and chemical inhibition of IRAK4 result in metabolic and immune improvements in high-fat diets. Remarkably, our results suggest that TMA—unlike its liver co-metabolite trimethylamine *N*-oxide, which is associated with cardiovascular disease—improves immune tone and glycemic control in diet-induced obesity. Altogether, this study supports the emerging role of the kinome in the microbial–mammalian chemical crosstalk.

## Abstract (web summary)

The microbial metabolite trimethylamine (TMA), the precursor of TMAO, which is associated with adverse cardiometabolic outcomes, is shown to have beneficial metabolic and anti-inflammatory effects in the host in the context of obesity.

## Main

Globally, diabetes affects 529 million individuals^1^, claiming 1.6 million lives per year according to the World Health Organization. IR is a multifactorial condition and is now increasingly common owing to, among other factors, the increase in prevalence of a sedentary lifestyle, Western-style foods and obesity. IR is a risk factor for developing type 2 diabetes and cardiovascular diseases^2^. One of the hallmarks of these disorders is the early development of chronic, systemic, low-grade inflammation^3^. The interaction between a high-fat diet (HFD) and the gut microbiome has a strong impact on the onset of IR^4–6^: bacterial lipopolysaccharides (LPS) and dietary fats trigger low-grade inflammation^2^ through activation of Toll-like receptor 4 (TLR4), a process called metabolic endotoxemia^7^.

This process is supported by complex communication that occurs between the gut microbiota and the innate immune system^8^, with consequences on metabolic homoeostasis^9^. Although some of the functional signalling molecules mediating microbial–mammalian chemical crosstalk have been characterized, a limited number of metabolite classes and their targets have been identified (G-protein-coupled^10^ and nuclear receptors^11^). However, it has been hypothesized that microbial metabolites potentially affect other pharmacological target classes, such as kinases^12^. In previous studies, we and others have identified a family of microbiome-associated metabolites, methylamines, associated with IR, non-alcoholic fatty liver disease^13^ and atherosclerosis^14^, but their mechanisms of action on mammalian hosts remain unclear.

TMA is one of the most abundant microbial metabolites produced by the gut microbiota. Previously, we reported that TMA may be associated with IR^12^. TMA results from microbial metabolism of dietary choline, carnitine and trimethylamine *N*-oxide (TMAO)^15–19^ before being absorbed and *N*-oxidized into TMAO by hepatic flavin-containing mono-oxygenase 3 (FMO3)^20^. After initial reports associating TMAO with adverse cardiovascular outcomes^13,16^, it has since emerged that FMO3 inactivation was beneficial for several metabolic outcomes^21–24^, strongly suggesting that TMA and TMAO have distinct biological roles.

Here, we decipher the role of TMA in the microbiota–host kinome chemical crosstalk in IR through (1) identification of gut-derived microbial metabolites associated with HFD-induced impaired glucose tolerance (IGT), IR and obesity; (2) pharmacological target screening of discriminant microbial metabolites; and (3) mechanistic validation of the pathophysiological relevance of pharmacological interactions with in vitro and in vivo models. Using this approach, we discovered a mechanism by which gut microbial TMA acts as an IRAK4 inhibitor and directly improves the host immune and metabolic tone.

### Lack of hepatic inflammation in HFD and the choline supplementation hypothesis

**Fig. 1:**
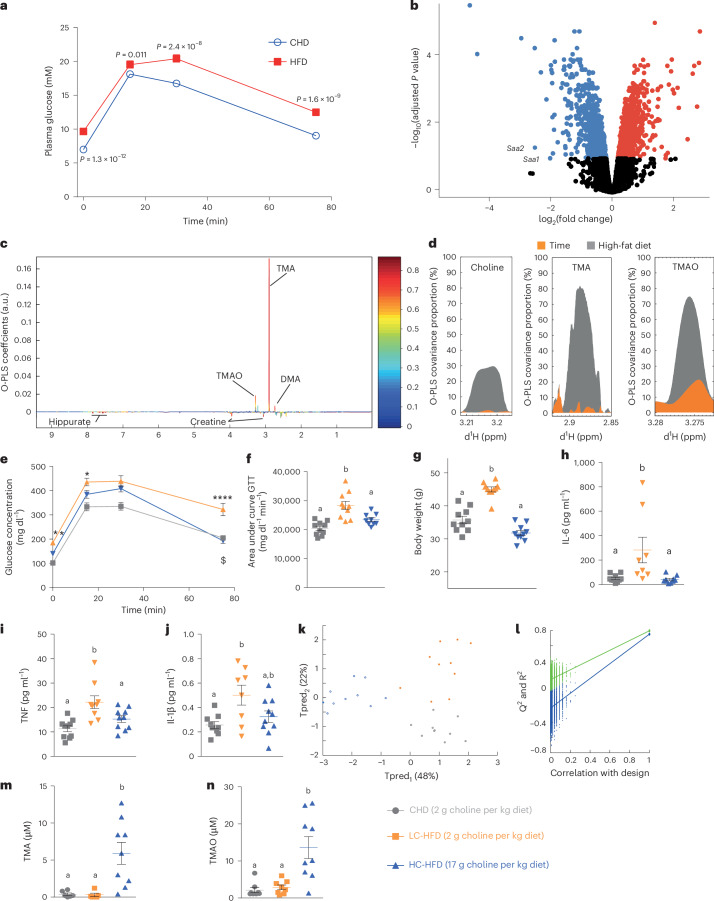
Choline supplementation improves glucose homoeostasis and inflammation after 5 months of HFD. Male mice were weaned at 3 weeks and fed a CHD diet before being randomly assigned to a CHD group (*n* = 68) or HFD group (*n* = 47) and monitored at 5 months of age. **a**, Effect of HFD feeding on GTT. **b**, Volcano plot of differentially expressed liver genes, highlighting that *Saa1* and *Saa2* are downregulated in HFD after 4 months of HFD. Red dots, significantly upregulated genes (false discovery rate (FDR) < 0.1); blue dots, significantly downregulated genes (FDR < 0.1). *P* values were corrected for multiple comparisons using the Benjamini–Hochberg method. **c**, Metabolic signature of diet on O-PLS model coefficient plot; line plot corresponds to covariance, and the colour scale represents proportion of variance explained. a.u., arbitrary units. **d**, Assessment of the contribution of factors diet and age on choline, TMA and TMAO by O-PLS variance components analysis: 5-week-old C57BL/6J mice were fed a CHD diet, a low-choline HFD or a low-choline HFD supplemented with choline (*n* = 10) and were phenotyped after 5 months. **e**, Plasma glucose concentration during an intraperitoneal GTT (ipGTT). **f**, Area under the curve of the plasma glucose concentration during an ipGTT. **g**, Body weight measured after 5 months of diet. **h**–**j**, Quantification of circulating cytokines: IL-6 (**h**), TNF (**i**) and IL-1β (**j**). **k**, Scores plot from a cross-validated O-PLS-DA model segregating the three groups of mice according to diet based on isotopic quantification of methylamines by UPLC–MS/MS; each component’s explained variance is shown in parentheses. **l**, Empirical assessment of the significance of O-PLS goodness-of-fit parameters by generating a null distribution with 10,000 random permutations. **m**,**n**, Quantification of circulating TMA (**m**) and TMAO (**n**) by UPLC–MS/MS. Data are means; error bars, s.e.m. One-way ANOVA followed by Tukey’s post hoc tests (superscript letters for factor levels, *P* < 0.05) on log-transformed data except **k** and **l**. Source data are provided. Source data

**Extended Data Fig. 1:**
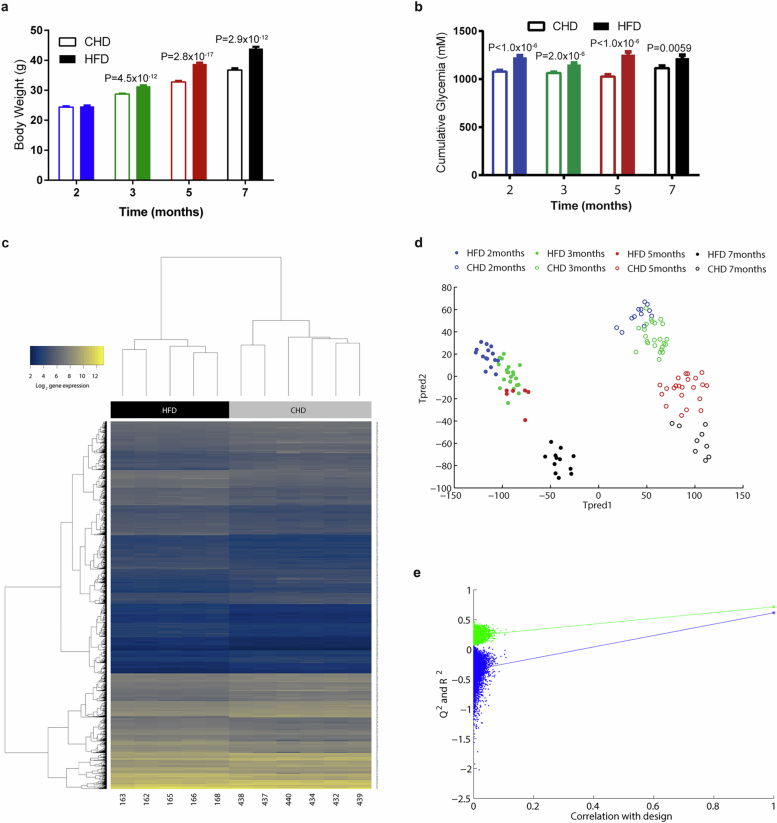
C57BL/6 mice fed HFD display low acute-phase gene expression in liver paralleled by high TMA excretion. Male mice were weaned at 3 weeks and fed CHD, before being randomly assigned to CHD group (n = 270) or HFD (n = 202) groups and monitored at 2 (72 CHD and 64 HFD), 3 (84 CHD and 57 HFD), 5 (68 CHD and 47 HFD) and 7 months (46 CHD and 34 HFD). (**a**, **b**) Effect of HFD-feeding on physiological parameters. (**a**) Body weight (BW) and (**b**) cumulative glycemia during GTT (AUC). (**c**) Heatmap of significantly (FDR < 0.1) differentially expressed liver genes after 4 months of HFD. (**d**) ^1^H-NMR-based metabotyping scores plot from a cross-validated O-PLS-DA model segregating the eight groups of mice according to both diet and age. (**e**) Empirical assessment of the significance of O-PLS goodness-of-fit parameters by generating a null distribution with 10,000 random permutations. Data are means ± s.e.m. Two-sided Mann-Whitney test (*P < 0.05 vs control). For the O-PLS-DA permutation test, the horizontal axis corresponds to the correlation between the original class membership variable (single dots on the right) and the randomly permuted class membership vectors (no longer correlated with the original class membership) (swarm of permutated models on the left). The *y* axis corresponds to goodness-of-fit parameter *R*^2^ (in green) derived for each O-PLS-DA model and the goodness-of-prediction parameter *Q*^2^ (in blue) derived by 7-fold cross-validation of each O-PLS-DA model. The *R*^2^ and *Q*^2^ parameters obtained from the original model in the top right corner are outside the confidence interval of the 10,000 randomly permuted models and therefore confirming the significance of the fitness and prediction capacity of the original O-PLS-DA model. Source data are provided. Source data

**Extended Data Fig. 2:**
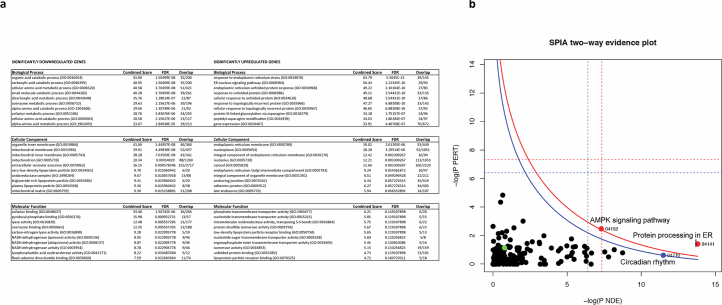
Pathway analysis for hepatic significant genes. (**a**) Gene ontology analysis for down- and up-regulated HFD-responsive genes. (**b**) Signaling pathway impact analysis (SPIA) for HFD-responsive genes was conducted as described^80^. Pathways respectively on the right-hand side of the red and blue curves are significant after Bonferroni correction of the global p-values (pG) obtained by combining the pPERT and pNDE using the normal inversion method, and significant after a FDR correction of pG using the Benjamini – Yekutieli method. Significant KEGG pathways: 04141, Protein processing in the endoplasmic reticulum; 04710, Circadian rhythm; 04152, AMPK signaling pathway. Source data are provided. Source data

We initially carried out longitudinal pathophysiological monitoring in a cohort of C57BL/6J mice (*n* = 5 for HFD and *n* = 6 for chow diet (CHD)), which rapidly developed obesity and IGT when fed a 65% kCal HFD with a range of macronutrient and micronutrient differences compared to CHD (Extended Data Fig. 1a,b, Fig. 1a and Supplementary Table 1). Liver transcriptomics (*n* = 5–6 per diet group) identified 2,084 significantly (false discovery rate of <0.1) differentially expressed genes between CHD-fed and HFD-fed mice (Extended Data Fig. 1c, Fig. 1b and Supplementary Table 1). Gene ontology and signalling pathway impact analyses (Extended Data Fig. 2 and Supplementary Tables 2–4) demonstrated upregulation of protein processing in the endoplasmic reticulum, whereas carbohydrate metabolism, circadian rhythm and AMPK signalling were significantly inhibited, consistent with existing literature^25,26^. Surprisingly, inflammation-associated pathways were not differentially affected: expression of genes coding for acute-phase serum amyloid A proteins, *Saa1* and *Saa2*, which are upregulated in response to acute inflammation and associated with type 2 diabetes, was reduced, whereas expression of pro-inflammatory cytokines such as *Il6* and *Il1β* was not significant (Fig. 1b and Supplementary Table 4). This is in contrast to established knowledge, as low-grade inflammation is one of the key features associated with IR^2,6^ and HFD^27^. In addition to the percentage of fat content as a macronutrient, we therefore looked for variation in micronutrients and microbial metabolites that may contribute to this phenotype by performing metabolic profiling of the urinary metabolome of this mouse cohort (CHD and HFD, four time-points) by proton nuclear magnetic resonance (^1^H-NMR) spectroscopy, followed by multivariate modelling. Our analysis showed that diet is the main factor influencing the metabolic profiles, followed by age (Extended Data Fig. 1d), with the model’s goodness-of-prediction parameters being highly significant upon permutation testing (Extended Data Fig. 1e). The urinary metabolic signature of HFD feeding compared to CHD was mainly associated with TMA excretion (Fig. 1c), consistent with our previous reports using this diet^13,28^. We used variance components analysis to decompose the contribution of diet and age to the excretion of these three metabolites, showing that the HFD contribution overwhelms the contribution of age (Fig. 1d). Given that the HFD contains >15 g of choline per kg of diet, we then investigated whether supplementation of this micronutrient could modulate the metabolic and immune variation in response to HFD feeding.

### Choline supplementation corrects HFD-induced inflammation and IR

**Extended Data Fig. 3:**
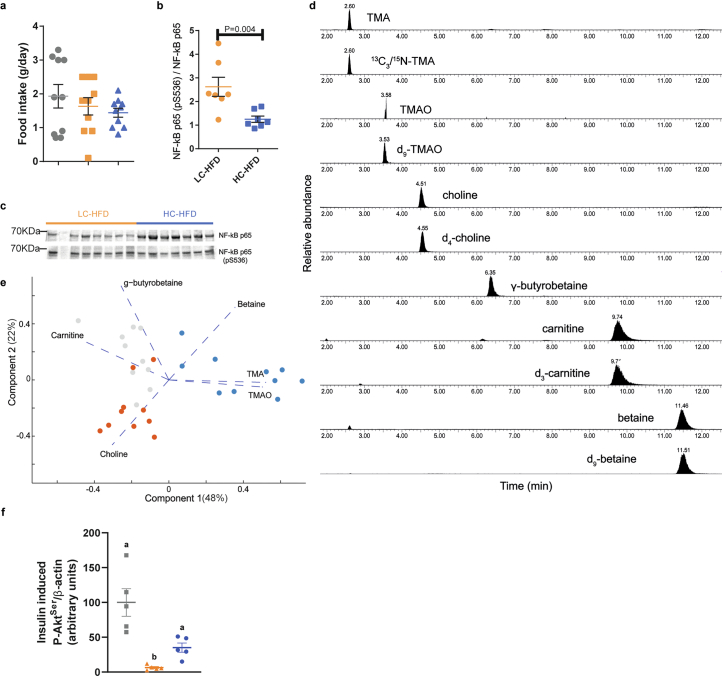
Choline supplementation corrects HFD adverse effects and associated methylamine measurements. (**a**) Food intake measured on the three tested diets (CHD, LC-HFD and HC-HFD), with each dot representing a single mouse. (**b**, **c**) Western blot analysis of liver NF-kB phosphorylation state between LC-HFD and HC-HFD (N = 7 per diet). In (**b**) each dot represents densitometric analysis of NF-κB^Ser536^ phosphorylation in liver extracts of a single mouse from the blot in (**c**). (**d**) Plasma sample chromatogram for the simultaneous detection of TMA, TMAO, choline, γ-butyrobetaine, carnitine, betaine and their isotopically labelled analogues. (**e**) Principal Component Analysis Biplot of plasma methylamine quantifications by UPLC/MS-MS from mice fed CHD, LC-HFD and HC-HFD, each component’s explained variance is shown in parenthesis. Data are means ± s.e.m. Two-sided Mann-Whitney test (*P < 0.05 vs control). (**f**) Densitometric analyses of western blots in Fig. 2g using β-actin to correct Akt levels prior to pAkt normalization. Each data point represents pAkt^Ser473^/β-actin ratio in liver extracts of a single mouse. Data are means ± s.e.m. One-way ANOVA followed by Tukey’s *post hoc* tests (superscript letters for factor levels *P* < 0.05) on log-transformed data. Source data are provided. Source data

To test the effect of choline supplementation itself on glucose tolerance and IR in an HFD context, we initiated a series of in vivo studies. We first fed C57BL/6J mice with a 65% kCal HFD containing 2 g kg^−1^ or 17 g kg^−1^ of choline (that is, LC-HFD and HC-HFD, respectively; Fig. 1e–n). To evaluate metabolic homoeostasis, we performed glucose tolerance tests (GTTs), which displayed a similar pattern with normalization of cumulative glycemia on HC-HFD compared to LC-HFD (Fig. 1e,f). Body weight was significantly increased in mice fed a LC-HFD from weaning until 5 months of age compared to mice fed a CHD and, remarkably, compared to mice fed HC-HFD (Fig. 1g), which was not related to food intake (Extended Data Fig. 3a). We then profiled circulating pro-inflammatory cytokines such as IL-6, IL-1β and TNF (Fig. 1h–j) and phosphorylation of NF-κB regulating their transcription (Extended Data Fig. 3b,c), which was also suggestive of an improvement of HFD-induced inflammation by choline supplementation.

### Targeted analysis of choline and methylamine pathways

To document the effect of choline supplementation on choline-related metabolic pathways, we further refined our isotopic quantification using ultra-performance liquid chromatography with tandem mass spectrometry (UPLC–MS/MS)^29,30^ to evaluate plasma concentrations of choline-derived and carnitine-derived metabolites leading to TMA and TMAO (Extended Data Fig. 3d,e). An orthogonal partial least squares discriminant analysis (O-PLS-DA) model significantly segregates the three treatment groups at 5 months of age (that is, 4 months of HFD) and highlights a choline supplementation effect on the first predictive component (Fig. 1k,l). Quantifications all reflect an increase in TMA and TMAO in the HC-HFD in line with choline supplementation (Fig. 1m,n). In particular, the circulating TMA levels were similar in the CHD and the LC-HFD (0.38 µM in CHD vs 0.3 µM in LC-HFD) and were about 20 times lower than in the HC-HFD (5.9 µM). These results depict an increased microbial conversion of choline into TMA in our HC-HFD, an observation already made in a previous study^13^. These results suggest that TMA could mediate the metabolic and immune benefits of choline supplementation.

### Baseline metabolic phenotyping after 2-month choline modulation in HFD

**Fig. 2:**
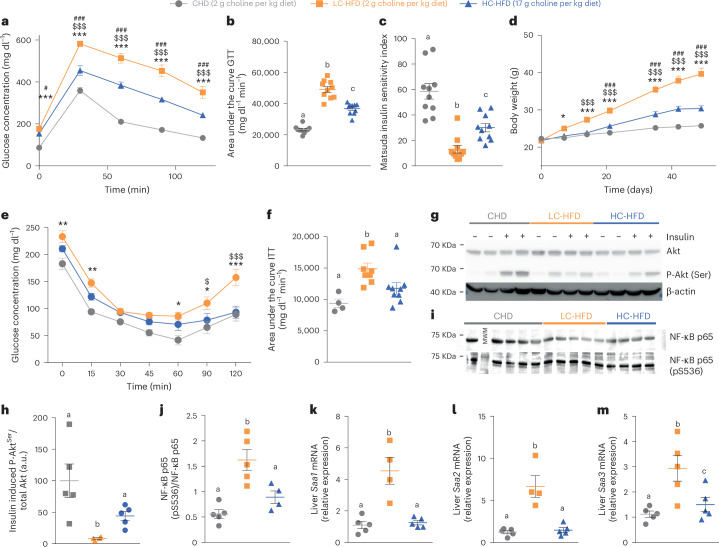
Choline supplementation corrects HFD adverse effects on glucose homoeostasis, insulin sensitivity and inflammation after 8 weeks of diet. C57BL/6J mice (5 weeks old) were fed CHD, LC-HFD or LC-HFD supplemented with choline (*n* = 10) and then phenotyped after 8 weeks. **a**, Plasma glucose concentration during an ipGTT. **b**, Area under the curve of the plasma glucose concentration during an ipGTT. **c**, Matsuda insulin sensitivity index calculated from the ipGTT. **d**, Body weight measured periodically during 8 weeks of diet. **e**, Plasma glucose concentration during an ipITT. **f**, Area under the curve of the plasma glucose concentration during an ipITT. **g**–**j**, Western blot analysis of liver Akt phosphorylation (**g**,**h**) and NF-kB phosphorylation (**i**,**j**). **k**–**m**, Expression of hepatic acute-phase inflammation proteins markers *Saa1* (**k**), *Saa2* (**l**) and *Saa3* (**m**). Data are means; error bars, s.e.m. In **c**, **d**, **f** and **h**–**m**, one-way ANOVA followed by Tukey’s post hoc tests (superscript letters for factor levels, *P* < 0.05) on log-transformed data. In **a**, **b** and **e**, a non-parametric two-sided Mann–Whitney test was used for each time-point; significance comparison signs are as follows: (*) CHD vs LC-HFD; ($) LC-HFD vs HC-HFD; (#) CHD vs HC-HFD. Source data are provided. Source data

To further assess the impact of choline supplementation on metabolic homoeostasis and low-grade inflammation in HFD contexts, we performed a second experiment, feeding C57BL/6J mice a 60% kcal HFD with a time frame comparable to those used in subsequent experimental designs using osmotic minipumps for constant subcutaneous TMA delivery. GTTs and weekly body weights showed a similar trend after 8 weeks of HFD feeding (Fig. 2a–d). Choline supplementation not only improved glucose tolerance (Fig. 2a,b) but also the Matsuda Index (Fig. 2c), a marker for IR derived from oral GTTs^31^. The normalization of insulin sensitivity was confirmed through insulin tolerance tests (ITTs) and insulin-induced hepatic Akt phosphorylation assays (Fig. 2e–h and Extended Data Fig. 3f for p-Akt/Akt ratio correction with β-actin). Notably, the normalization of insulin-induced hepatic AKT phosphorylation assays was primarily associated with a reduction of the basal level of p-Akt in HC-HFD compared to LC-HFD observed in saline conditions, demonstrating the reduction of IR (Fig. 2g). Indeed, it has been previously demonstrated that HFD increased the basal levels of p-Akt, and this contributes to IR^32^. We further characterized that HFD feeding increased hepatic NF-κB phosphorylation compared to CHD, which was normalized by choline supplementation, although this could also be a result of variable NF-κB protein levels between samples (Fig. 2i,j), a pattern that was also observed for expression of acute-phase proteins that are typically upregulated in response to tissue inflammation^33^ (*Saa1*, *Saa2* and *Saa3*; Fig. 2k–m).

### Chemical blockage of bacterial TMA biosynthesis and loss of metabolic benefits

**Fig. 3:**
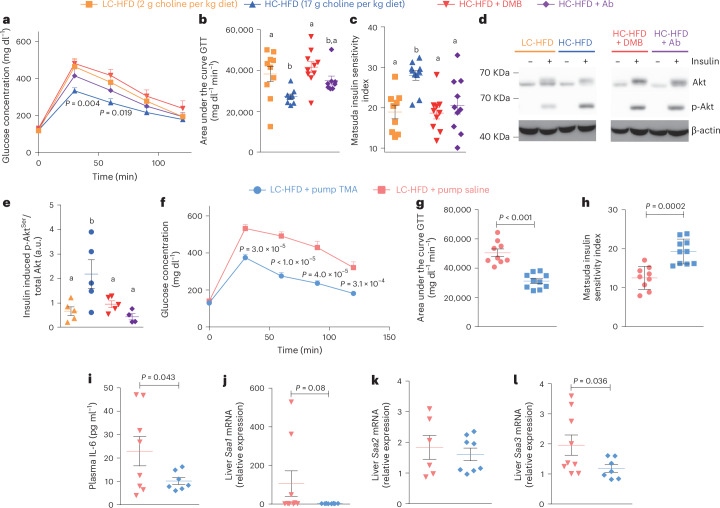
Blocking TMA production from choline cancels metabolic benefits from choline supplementation, and chronic TMA treatment mimics it. **a**–**e**, C57BL/6J mice (5 weeks old) were fed LC-HFD or LC-HFD supplemented with choline (*n* = 10). Two strategies were used in parallel to block TMA production from choline by the gut microbiota, using either 1% DMB in the diet or an antibiotic treatment. **a**, Plasma glucose concentration during an ipGTT. **b**, Area under the curve of the plasma glucose concentration during an ipGTT. **c**, Matsuda insulin sensitivity index calculated from the ipGTT. **d**,**e**, Western blot analysis of liver Akt phosphorylation. **f**–**l**, Mice were weaned at 3 weeks and fed a LC-HFD before being fitted with osmotic minipumps delivering a chronic circulating dose of TMA (0.1 mM) for 6 weeks (*n* = 9 saline HFD, *n* = 8 TMA HFD), with each data point representing a single mouse. **f**, Plasma glucose concentration during an ipGTT. **g**, Area under the curve of the plasma glucose concentration during an ipGTT. **h**, Matsuda insulin sensitivity index calculated from the ipGTT. **i**, Quantification of circulating IL-6. **j**–**l**, Expression of hepatic acute-phase inflammation protein markers *Saa1* (**j**), *Saa2* (**k**) and *Saa3* (**l**). Data are means; error bars, s.e.m. Results were assessed by one-way ANOVA followed by Tukey’s post hoc tests (superscript letters for factor levels, *P* < 0.05) on log-transformed data. Source data

**Extended Data Fig. 4:**
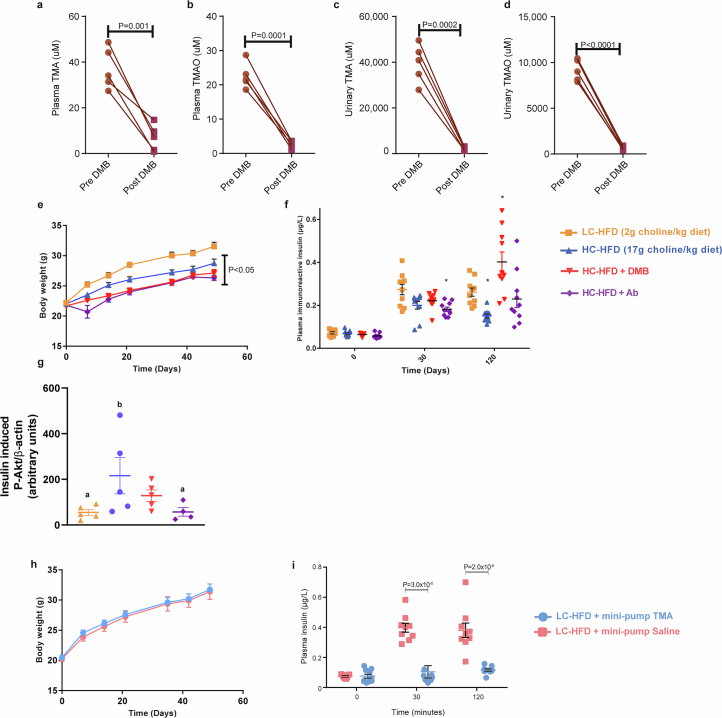
Modulation of TMA production and effects. (**a**–**d**) Confirmation of blockage of production of TMA (**a**, **c**) and TMAO (**b**, **d**) by 1% DMB measured in plasma (**a**, **b**) and urine (**c**, **d**). Each line represents TMA or TMAO levels trajectories pre (circles) or post (squares) DMB treatment in a single mouse (**e**) Body weight monitoring during the whole experiment of mice fed a LC-HFD and a HC-HFD supplemented or not with DMB or treated with antibiotics. (**f**) Plasma insulin concentration during GTT at 0, 30 and 120 min post glucose injection. (**g**) Densitometric analyses of western blots in Fig. 3d using β-actin to correct Akt levels prior to pAkt normalization. One-way ANOVA followed by Tukey’s *post hoc* tests (superscript letters for factor levels *P* < 0.05) on log-transformed data. For (e-g) each dot represents data from a single mouse. (**h**) Body weight monitoring during the whole experiment of mice fed a LC-HFD and chronically treated with TMA (N = 10; light blue) or saline (N = 10; light red). (**i**) Plasma insulin concentration during GTT at 0, 30 and 120 min post glucose injection. Each data point represents data from a single mouse. Data are means ± s.e.m. Two-sided Mann-Whitney test (*P < 0.05 vs LC-HFD control). Source data are provided. Source data

To test whether the beneficial effects of choline supplementation are mediated by its microbial product TMA, we sought to block bacterial TMA production in HC-HFD-fed mice both non-specifically and specifically using a wide-spectrum antibiotics cocktail or 3,3-dimethyl-1-butanol (DMB), inhibiting microbial TMA-lyase^34^, respectively. We first confirmed the functional blockage of TMA production in mice fed a HC-HFD, resulting in a drastic drop in circulating and excreted TMA and TMAO following 1% DMB administration (Extended Data Fig. 4a–d). In accordance with our hypothesis, both antibiotic and DMB treatments abolished the effects of choline supplementation-induced improvements in HFD, in particular for glucose tolerance, insulin sensitivity (as suggested by the Matsuda index; Fig. 3a–c and Extended Data Fig. 4e,f) and hepatic insulin sensitivity, as shown by the absence of a beneficial effect of choline supplementation on insulin-induced Akt phosphorylation (Fig. 3d,e and Extended Data Fig. 4g for β-actin normalization). The metabolic improvements associated with chemical TMA synthesis blockage were not a result of changes in body weight gain (Extended Data Fig. 4e), thereby strongly suggesting that the effects on glucose metabolism induced by inhibiting bacterial TMA production in HC-HFD were decoupled from obesity.

### TMA treatment mimics choline supplementation

To further confirm whether TMA mediates the beneficial effects of choline supplementation, we chronically treated LC-HFD-fed C57BL/6J mice with TMA for 6 weeks using subcutaneous osmotic minipumps and assessed their immunometabolism. We confirmed that chronic TMA treatment at 0.1 mM in a LC-HFD did not affect body weight gain (Extended Data Fig. 4h) but effectively normalized glucose homoeostasis and the Matsuda Index, a readout of insulin sensitivity (Fig. 3f–h and Extended Data Fig. 4i). TMA treatment also improved the pro-inflammatory response to HFD feeding and hepatic expression of acute-phase proteins *Saa1*, *Saa2* and *Saa3* (Fig. 3i–l). These results suggest that chronic TMA treatment decouples body weight gain and adiposity from low-grade inflammation and glucose homoeostasis, thereby mimicking the immune and metabolic benefits observed in choline supplementation.

### TMA is an IRAK4 kinase inhibitor

**Fig. 4:**
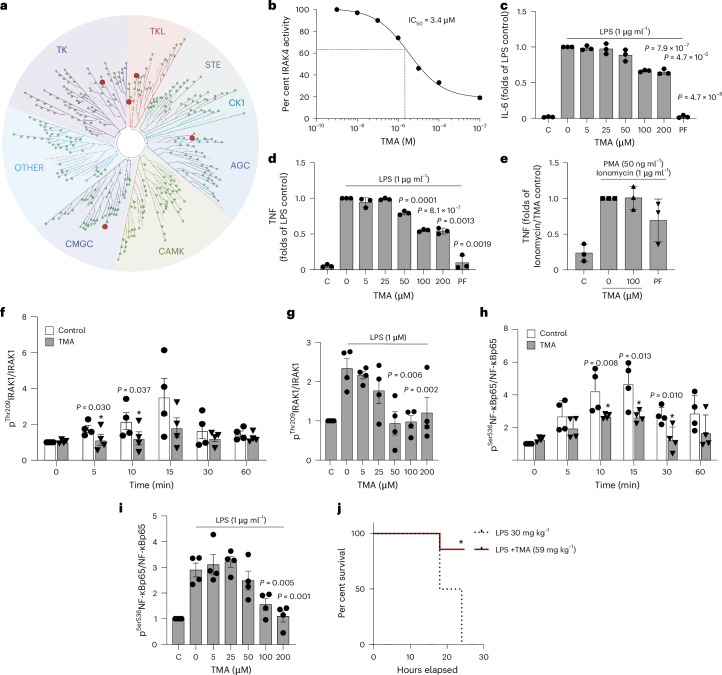
TMA inhibits IRAK4 and rescues LPS-induced TLR4-mediated pro-inflammatory response. **a**, Kinome screen for a single dose of TMA (0.1 mM) against 456 kinases. The kinases covered by the kinome scan assay are visualized using a phylogenetic layout. Preliminary positive hits with potential binding >35% vs control (DMSO) are represented by a red dot; non-binding kinases are represented by a green dot. Kinase group names: TK, tyrosine kinases; TKL, tyrosine kinase-like; STE, STE kinase group; CK1, cell linase 1; AGC, protein kinase A, G and C families; CAMK, calcium and calmodulin-regulated kinases; CMGC, CMGC kinase group. **b**, Functional characterization of the inhibition of IRAK4 by TMA. IRAK4 phosphorylation activity was determined in the presence of various concentrations of TMA and resulted in an IC_50_ of 3.4 µM. **c**,**d**, TMA preincubation (100 µM for 30 min) suppresses LPS-induced (1 µg ml^−1^, 4 h) IL-6 (**c**) and TNF (**d**) release by human PBMCs. The values of the unstimulated controls were arbitrarily set to 1. For **c** and **d**, statistical significance (*P* < 0.05) was assessed with a two-sided unpaired Student’s *t-*test (vs LPS-stimulated control). **e**, TMA (100 μM) or the IRAK4-specific inhibitor PF06650833 (50 nM) do not inhibit TNF secretion from human PBMCs after 4 h stimulation with PMA (50 ng ml^−1^) and ionomycin (1 μg ml^−1^). **f**, Impact of TMA pre-treatment (100 µM, 30 min) on human PBMCs’ ^Thr209^IRAK1/IRAK1 phosphorylation upon LPS (1 µg ml^−1^) stimulation for the indicated times. **g**, Human PBMCs were pre-treated (for 30 min) with the indicated concentrations of TMA, and ^Thr209^IRAK1/IRAK1 phosporylation was assessed after 10 min of LPS (1 μg ml^−1^) challenge. The time-dependent (**h**) and dose-dependent (**i**) effect of TMA on phosphorylation of ^Ser536^NF-κBp65/NF-κBp65 of human PBMCs stimulated with LPS (1 μg ml^−1^) was assessed as in **f** and **g**. For **f**–**i**, the control value was arbitrarily set to 1, and statistical significance (*P* < 0.05) was assessed with a one-sided unpaired Student’s *t-*test. For PBMC experiments in **c**–**i**, each data point represents a separate experiment. **j**, A 24 h Kaplan–Meier survival curve of mice challenged with a lethal dose of LPS (30 mg kg^−1^) that received a single dose of TMA (59 mg kg^−1^, red line), or vehicle (black line); *P = 0.0047 determined by two-sided log-rank Mantel–Cox test. Data are means; error bars, s.e.m. Source data

**Extended Data Fig. 5:**
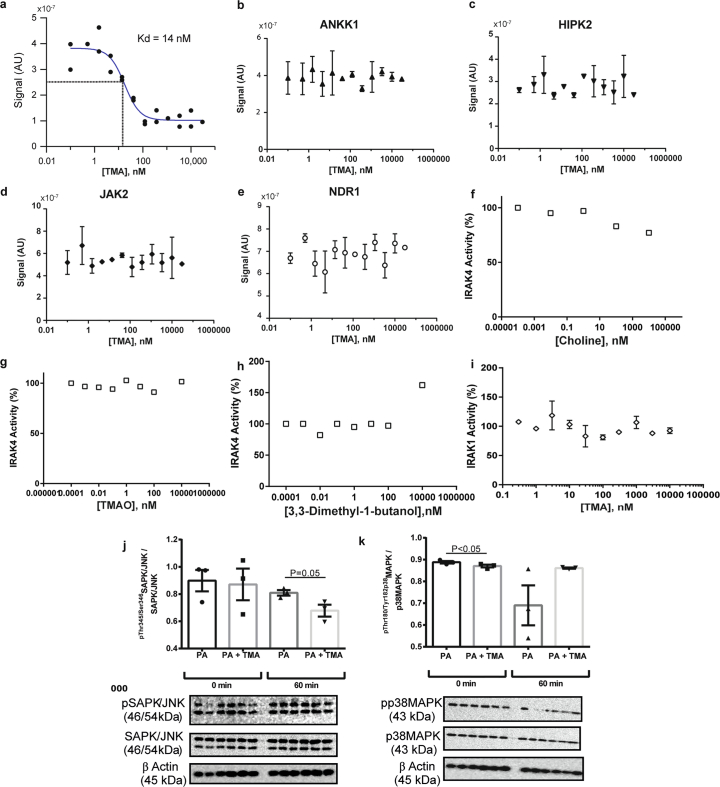
TMA does not bind with kinases ANKK1, HIPK2, JAK2, and NDR1 identified as candidates in the kinome screen, but makes significant contact with IRAK4. (**a**) Confirmation of a physical interaction between TMA and IRAK4 using TMA at concentrations ranging from 0.1 nM to 100 µM, resulting in a dissociation constant (*K*_*d*_) of 14 nM. (**b**–**e**) Physical interaction test between TMA and the four hits identified using a kinome screen, using TMA concentrations ranging from 0.1 nM to 100 µM for (**b**) ANKK1, (**c**) HIPK2, (**d**) JAK2 and (**e**) NDR1. For (b-e) N = 2 biological repeats. IRAK4 kinase activity is not significantly inhibited by choline (**f**), TMAO (**g**) or DMB (**h**), while TMA does not have any inhibitory effect on IRAK-1 kinase activity (average of N = 2 biological repeats) (**i**). (**j**, **k**) Phosphorylation for ^pThr183/Tyr185^SAPK/JNK/SAPK/JNK (**j**) and ^pThr180/Tyr182^p38MAPK/p38MAPK (**k**) densitometric ratios, represented by each dot. Data are means ± s.e.m. One-way ANOVA followed by Tukey’s *post hoc* tests (superscript letters for factor levels *P* < 0.05) on log-transformed data. Source data are provided. Source data

To identify a direct mechanism linking TMA to metabolic and immune benefits in HFD-fed mice, we sought to identify its host pharmacological targets. The kinome, made of 518 kinases encoded in the human genome^12^, represents a repertoire of critical signal transduction switches for metabolic homoeostasis and inflammation. To discover specific physical interactions, we screened TMA against a panel of 456 clinically relevant human kinases using a high-throughput assay^35,36^ (see Methods) and identified five preliminary hits for TMA (Fig. 4a and Supplementary Table 5). We then generated multiple-dose binding curves between TMA and each hit and confirmed that TMA physically binds IRAK4 (dissociation constant (*K*_*d*_) = 14 nM; Extended Data Fig. 5a) but not the other four preliminary hits (flat dissociation curves with no *K*_*d*_ fit in Extended Data Fig. 5b–e, suggesting no physical binding was observed). Given that *K*_*d*_ only addresses a physical interaction in its simplest form (that is, binding), we functionally tested TMA as an IRAK4 inhibitor by quantifying IRAK4 kinase activity in the presence of ATP and increasing doses of TMA (see Methods) to derive a half-maximal relative inhibitory constant (IC_50_ = 3.4 µM; Fig. 4b) and an inhibitory constant *K*_i_= 0.7 µM (see Methods). We also confirmed that choline, TMAO and DMB do not inhibit IRAK4 kinase activity (Extended Data Fig. 5f–h), and further verified that TMA does not inhibit IRAK1 (Extended Data Fig. 5i), as suggested by the phenotypic convergence of *Irak1*^−/−^ mice fed a HFD, which were shown to have metabolic improvements similar to TMA treatment^37^. Nonetheless, this screen, combined with purified kinase activity assays, suggests that IRAK4 is the specific kinase target for TMA, which we next validated using a range of in vitro and in vivo experiments.

### TMA inhibits IRAK4 signalling in human PBMCs and primary human hepatocytes

IRAK4 is a central kinase in the TLR pathway, sensing bacterial invasion and promoting a pyretic pro-inflammatory response in infectious contexts^38,39^. To further corroborate the inhibitory action of TMA on IRAK4 on innate immunity, we studied its effect on human peripheral blood mononuclear cell (PBMC) cytokine secretion and NF-κB signalling in response to LPS stimulation, a process exclusively mediated by IRAK4 downstream of TLR4, as previously demonstrated^38^. Consistent with this previous work, pre-treatment with TMA for 30 min dose-dependently blunted IL-6 and TNF secretion by PBMCs challenged with LPS after 4 h (Fig. 4c,d). Moreover, IL-6 and TNF secretion was virtually abolished by pre-treatment with the IRAK4-specific inhibitor PF06650833 (ref. ^40^) as an additional control (100 nM). Conversely, TMA (100 µM) or the IRAK4-specific inhibitor PF06650833 (100 nM) had no significant impact on TNF secretion by PBMCs stimulated by phorbol-ester/Ionomycin (Fig. 4e), similarly to human B cells harbouring IRAK4-inactivating mutations^41^. Taken together, these findings support our assertion that TMA acts through IRAK4 and further validate the functional inhibition of IRAK4 by TMA in innate immune cells. In addition, TMA (0.1 mM) significantly reduced IRAK1 phosphorylation at a site (Thr209) phosphorylated by IRAK4 (ref. ^42^) upon short-term stimulation of PBMCs with LPS (up to 60 min; Fig. 4f). TMA also inhibited ^Thr209^IRAK1 phosphorylation dose-dependently after LPS stimulation of PBMCs (Fig. 4g) and, similarly, TMA time-dependently and dose-dependently suppressed NF-κBp65 phosphorylation in LPS-challenged PBMCs (Fig. 4h,i). In agreement with the cellular PBMC experiments, a single TMA dose significantly improved mouse survival in a 24 h LPS septic shock experiment (Fig. 4j), showing similar protection to IRAK4 kinase-inactive mice^43^.

We then sought to test IRAK4 inhibition in a HFD context. The main free fatty acid in our HFD is palmitate, a saturated free fatty acid triggering TLR4 signalling. Therefore, we used a primary human hepatocyte model of low-grade inflammation and IR^44^.

**Extended Data Fig. 6:**
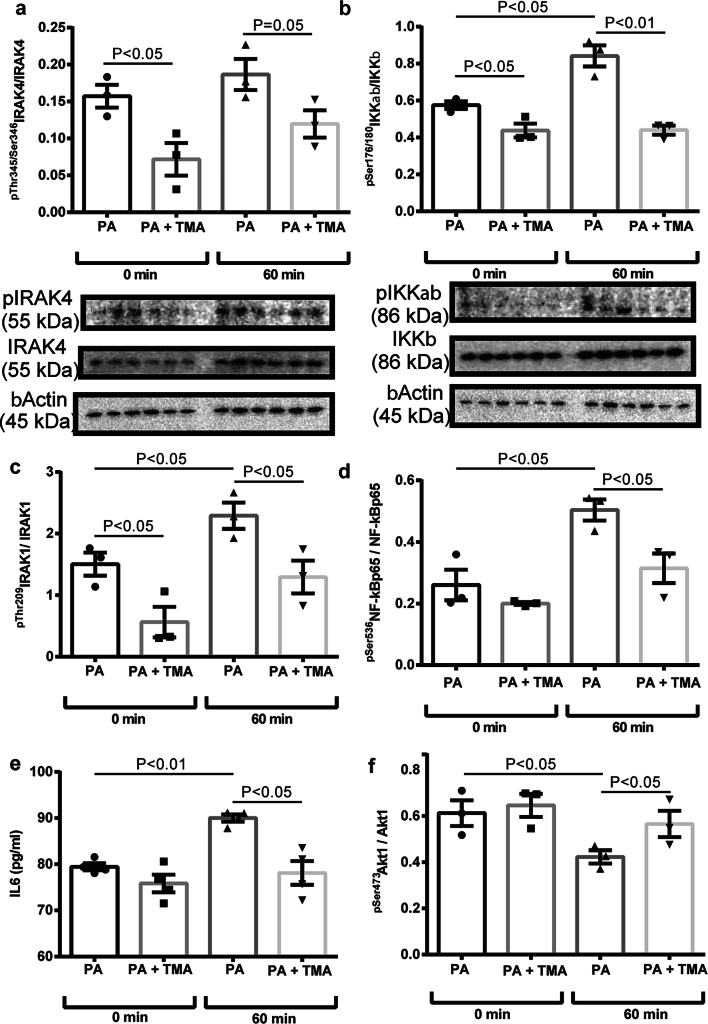
TMA inhibits IRAK4 and suppresses TLR4-mediated pro-inflammatory response in primary human hepatocytes. (**a**–**d**) Effect of palmitate (PA; 200 μM) administration and TMA pre-treatment (0.1 mM, 30 min) in primary human hepatocytes at 0 and 60 min on ^pThr345/Ser346^IRAK4/IRAK4 (**a**), ^pSer176/180^IKKαβ/IKKβ (**b**), ^pThr209^IRAK1/IRAK1 (**c**), ^pSer536^NF-κBp65/NF-κBp65 (**d**) ratios. For (a-b) each dot represents densitometric ratios of relative phosphorylation levels from hepatocyte lysates from 3 independent biological repeats, treated as indicated. ^*^p < 0.05 and ^**^p < 0.01 *vs* 0 min; ^†^p < 0.05 and ^††^p < 0.01 *vs* vehicle; ^b^p = 0.07 *vs* vehicle. Effect of PA (200 μM) administration (4 h) with and without TMA pre-treatment (0.1 mM, 30 min) on IL6 accumulation in hepatocyte media (**e**) and on ^pSer473^Akt1/Akt1 after insulin stimuli (100 nM, 10 min) (**f**). Each dot represents an independent biological repeat. *p < 0.05 and **p < 0.01 *vs* control-vehicle; †p < 0.05 *vs* control-PA. For (a-f) data are means ± s.e.m and statistical significance was determined by one-way ANOVA followed by Tukey’s *post hoc* tests on log-transformed data. Source data are provided. Source data

Under basal conditions (0 min), TMA (0.1 mM, 30 min) pre-treatment resulted in a significant decrease in p^Thr345/Ser346^IRAK4/IRAK4, p^Thr209^IRAK1/IRAK1 and p^Ser176/180^IKKαβ/IKKβ. Palmitic acid (200 μM, 60 min) administration resulted in increased p^Thr209^IRAK1/IRAK1, p^Ser176/180^IKKαβ/IKKβ and p^Ser536^NF-κBp65/NF-κBp65 (Extended Data Fig. 6a–d), which were normalized by TMA treatment. The TMA effect was significant as determined by a 2-way ANOVA for p^Thr345/Ser346^IRAK4/IRAK4 (*P* = 0.04), p^Thr209^IRAK1/IRAK1 (*P* = 0.03) and p^Ser176/180^IKKαβ/IKKβ (*P* < 0.001). Normalization of the phosphorylation ratios in the TLR4 pathway by TMA resulted in a normalization of palmitic acid-induced IL-6 secretion in the cell medium after 4 h (Extended Data Fig. 6e). TMA also prevented the negative impact of palmitic acid (200 μM, 4 h) on insulin action (specifically on insulin-induced p^Ser473^Akt1/Akt1), indicating a beneficial effect of TMA on insulin signalling (Extended Data Fig. 6f). TMA also tended to decrease p^Thr183/Tyr185^SAPK/JNK/SAPK/JNK induced by palmitic acid (200 μM, 60 min; Extended Data Fig. 5j) but had no significant effect on p38MAPK (Extended Data Fig. 5k). Collectively, our results demonstrate that TMA is an IRAK4 kinase inhibitor ameliorating LPS inflammatory signalling in PBMCs and mice and normalizing palmitate-induced low-grade inflammation and aberrant insulin signalling in vitro, requiring further assessment in *Irak4*^−/−^ mice.

### *Irak4*^−/−^ mice are protected against HFD-induced immune and metabolic dysregulations

**Fig. 5:**
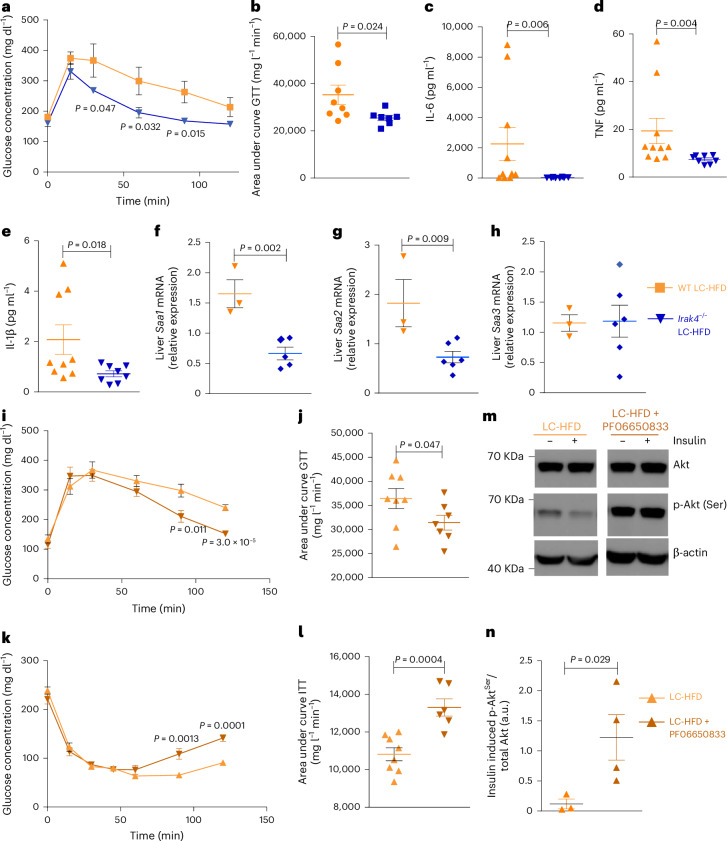
IRAK4 deficiency corrects IGT and pro-inflammatory response after 8 weeks of LC-HFD feeding in C57BL/6J mice, and a chemical inhibitor of IRAK4 mimics the effects on glucose homoeostasis. **a**–**h**, *Irak4*^−/−^ mice and wild-type (WT) littermates (5 weeks old) were fed a LC-HFD (*n* = WT HFD and *n* = 8 *Irak4*^−/−^ HFD) and were phenotyped after 8 weeks. Each point represents data from a single mouse. **a**, Plasma glucose concentration during an ipGTT. Two-sided Student’s *t*-test (*P < 0.05). **b**, Area under the curve of the plasma glucose concentration during an ipGTT. **c**–**e**, Circulating cytokines IL-6 (**c**), TNF (**d**) and IL-1β (**e**). **f**–**h**, Liver mRNA expressions for *Saa1* (**f**), *Saa2* (**g**) and *Saa3* (**h**) genes. For **b**–**h**, data are means; error bars, s.e.m. Statistical significance (*P* < 0.05) was determined using a one-tailed Mann–Whitney test. **i**–**n**, Mice were weaned at 3 weeks and fed a LC-HFD before being fitted with osmotic minipumps delivering a chronic circulating dose of PF06650833 (50 nM) for 6 weeks. **i**, Plasma glucose concentration during an ipGTT. Each point represents data from a single mouse. **j**, Area under the curve of the plasma glucose concentration during an ipGTT. **k**, Plasma glucose concentration during an ipITT. **l**, Area under the curve of the plasma glucose concentration during an ipITT. **m**,**n**, Western blot analysis of liver Akt phosphorylation. A representative immune blot from *n* = 3 repeats is shown. Data are means; error bars, s.e.m. For **i**–**n**, *P* values were determined by one-sided unpaired Student’s *t-*test (*P* < 0.05 was deemed statistically significant). For **n**, the data were log transformed. Source data

**Extended Data Fig. 7:**
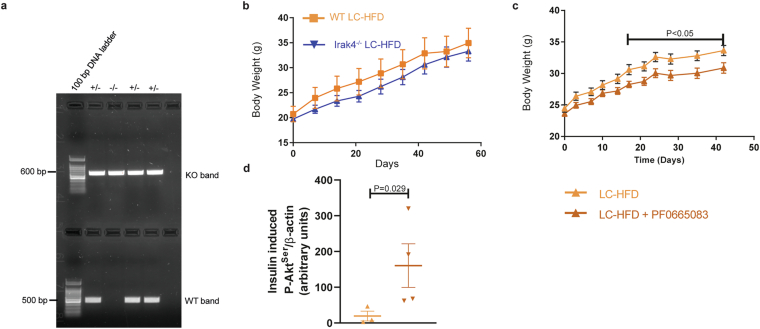
IRAK4 knock-out does not affect body weight while chronic treatment with PF06650833 reduces it in mice fed a low-choline HFD. (**a**) Indicative agarose gel of PCR products from +/-irak4 and -/-irak4 mice. (**b**, **c**) Body weight monitoring during the whole experiment of *Irak4*^−/−^ mice (N = 9) or wild-type controls (N = 9) (**b**) or PF06650833-treated (N = 9-13) or vehicle-treated (N = 6) mice (**c**) fed a LC-HFD. (**d**) Densitometric analyses of western blots in Fig. 5m using β-actin to correct Akt levels prior to pAkt normalization. Each data point represents pAKT^Ser473^/β-actin ratio from a single mouse liver. Data are means ± s.e.m. double-sided unpaired Student’s *t* test (*P < 0.05) on log-transformed data. Source data are provided. Source data

IRAK4 is a key kinase required for defence against pyogenic infections in acute contexts^38,39^. To further test whether this kinase has a role in HFD-induced chronic low-grade inflammation and glucose homoeostasis, we fed 5-week-old *Irak4*^−/−^ mice^45^ and wild-type littermates on a C57BL/6J background (Extended Data Fig. 7a for genotyping) a LC-HFD, to avoid potential confounding effects from TMA for 8 weeks before assessing circulating cytokines, expression of hepatic inflammatory genes and acute-phase markers and metabolic homoeostasis (Fig. 5a–h). *Irak4*^−/−^ mice presented improved glycemic control compared to wild-type littermates (Fig. 5a,b). Likewise, the inflammatory response to LC-HFD observed in wild-type mice was obliterated in *Irak4*^−/−^ littermates (Fig. 5c–g). There was a similar trend for *Saa3* (Fig. 5h), but there was no effect on body weight gains (Extended Data Fig. 7b). Hence, similarly to TMA treatment, genetic ablation of IRAK4 abolishes the HFD-induced pro-inflammatory response and IGT, thereby decoupling obesity from IGT and low-grade inflammation with a similar phenotype to *Irak1* deficiency^37^.

### Pharmacological inhibition of IRAK4 normalizes glucose metabolism

Given that the *Irak4*^−/−^ mice lack the whole protein, we compared the knockout phenotype with the phenotype of PF06650833, a recently discovered chemical inhibitor of the human IRAK4 protein^40^ that has shown promising results in a phase-I trial for rheumatoid arthritis^46^. Treatment with PF06650833 improved body weight in LC-HFD mice (Extended Data Fig. 7c). This inhibitor also yielded significant improvements in plasma glycemia at the latter time-points of the GTT and ITT, and in cumulative glycemia (Fig. 5i–l), which was mirrored by an increase in Akt phosphorylation (Fig. 5m,n and Extended Data Fig. 7d for β-actin normalization). These results collectively show that specific chemical inhibition of IRAK4 leads to significant improvements in glycemic control, insulin sensitivity and insulin signalling. Moreover, the data suggest that IRAK4 could constitute a clinically relevant target in IR and related disorders.

## Discussion

**Fig. 6:**
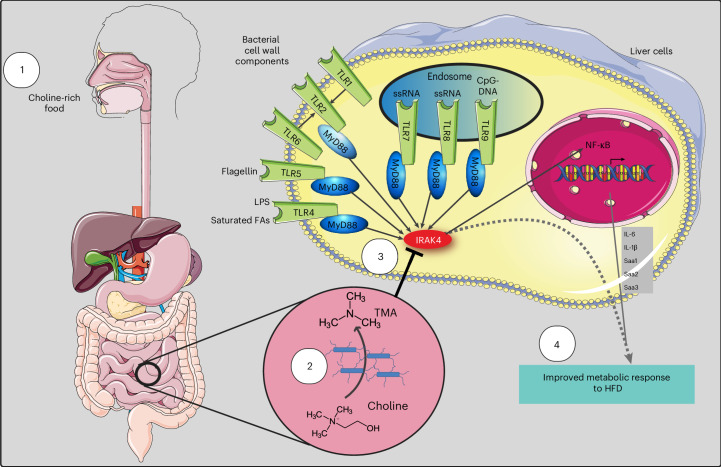
Overview of effects of IRAK4 inhibition by TMA on metabolic response to HFD. TMA is synthesized from dietary choline (1) by the gut microbiota (2) and specifically inhibits IRAK4 activity (3). This inhibition blunts the TLR signalling pathway (induced by LPS and saturated free fatty acids), leading to an improvement of metabolic response to HFD (for example, glucose homoeostasis). Image adapted from Servier Medical Art (https://smart.servier.com), licensed under CC BY 4.0 (https://creativecommons.org/licenses/by/4.0).

The discovery that TMA is a kinase inhibitor controlling IRAK4, a central kinase involved in innate immunity, is a major finding that provides an attractive mechanism for the metabolic and immune improvements observed with choline supplementation in HFD contexts (Fig. 6).

IRAK4 is the first regulatory checkpoint downstream of MyD88, the adaptor protein connecting IRAK4 to at least six TLRs sensing bacterial compounds or components^47^. IRAK4 deficiency is associated with bacterial infections in humans^39^ and in mice^48^. Consistent with the involvement of the TLR signalling pathway at the crossroads between gut microbiota and dietary lipids^49,50^, IRAK4 deletion and its inhibition by TMA and PF06650833 (ref. ^40^) rescued HFD-induced low-grade inflammation and IR^3,51,52^, highlighting new and unexpected roles for this microbial metabolite and its target kinase in immunometabolism. The relative IC_50_ (3.4 µM) that we determined is half the plasma isotopic quantifications obtained by UPLC–MS/MS in the HFD group supplemented with choline. These quantifications are comparable to circulating TMA levels previously reported in normal human plasma, ranging between 0.42 and 48 µM (refs. ^53,54^), which makes this IC_50_ particularly relevant with respect to pathophysiological mechanisms: dosing mice with 0.1 mM TMA was sufficient to improve inflammatory and metabolic responses. TMA can therefore be considered a ‘microbial signalling metabolite’^55^, sending a negative feedback signal to a pathway triggered by the influx of saturated free fatty acids in HFD contexts. This mechanism participates in maintaining a low immunological footprint and improved metabolic homoeostasis in a symbiotic relationship. The role of TMA as an IRAK4 inhibitor could, for instance, explain some of the beneficial effects reported for choline supplementation in patients with non-alcoholic fatty liver disease^56^ as well as the improved IR observed in those consuming higher choline diets in a healthy, genetically uniform human population^57^. Altogether, our results on IRAK4 inhibition and ablation provide further insight into the phenotypic convergence between *Myd88*, *Irak4* and *Irak1* knockout mouse models^37,58^, suggesting that the gut microbiota, through TMA, proceeds with a targeted ‘hijacking’ of the TLR signal transduction machinery to the host’s benefit, resulting in metabolic and immune improvements.

Our finding that TMA improves metabolic health in the context of HFD is surprising, given the extensive evidence linking higher levels of circulating TMAO (after phase-1 *N*-oxidation of TMA in the liver) with worse cardiovascular outcomes, particularly in patients with established atherosclerosis and thrombosis^14^. Our results show that the TMA-lyase inhibitor DMB blocks TMA production and abrogates the metabolic benefits brought about by choline supplementation in HC-HFD mice, suggesting that the benefits come from the conversion of choline to TMA.

Our findings are supported by the discovery that in diabetic mice, knockdown of the liver gene (*Fmo3*) that converts TMA to TMAO—an intervention that would increase TMA levels—improves IR and attenuates atherosclerotic burden^23^. Similarly, in an epidemiological study in a genetically homogeneous population, higher choline consumption was associated with improved insulin sensitivity^57^.

Our work is consistent with the idea that TMA and TMAO may well have contrasting roles that are context-dependent and mechanism-dependent.

To illustrate context dependence, TMAO has different effects based on the insult. It has been linked to poor cardiovascular outcomes in humans; however, independently of its role in cardiovascular disease, it increases kidney fibrosis^59^, beneficially reduces blood–brain barrier permeability^60^ and is associated with improved survival outcomes in septic ventilated patients^61^. Moreover, TMAO levels in healthy adults did not predict future coronary events in a 10-year follow-up study^62^, suggesting that in this context, TMAO might often require underlying pathology for its detrimental effects to become overt. Contrary to coronary heart disease, in which significant evidence points towards a detrimental role by TMAO^63^ for obesity-driven IR and dysregulated glucose handling (which is the main focus of this work), the role of TMAO is unclear, with conflicting human studies pointing to protective^28,64^, detrimental^65^ or insignificant^66,67^ TMAO effects. Mice fed a choline-enriched (1%) CHD showed IGT compared to those on a CHD and impaired β cell function, owing to increased plasma TMAO^68^, in an apparent contradiction to our results in HFD-challenged mice. We have demonstrated a similar diet-dependent phenomenon with the host-microbiome co-metabolite hippurate, whereby hippurate improved glucose handling and IR only in mice fed a HFD^69^. Similarly, TMA has shown context dependence, negatively affecting the blood–brain barrier^60^ and endothelial vasoconstriction^70^.

To illustrate the mechanism dependence, TMAO augments Ca^2+^-mediated platelet aggregation after thrombin treatment. TMAO also binds to and activates purified protein kinase RNA-like ER linase (PERK), while structurally related metabolites such as choline do not^71^. This proatherothrombic mechanism may well explain the adverse cardiovascular outcomes associated with TMAO^13,16^. Conversely, it has been shown that FMO3 inactivation, preventing *N*-oxidation of TMA and therefore increasing TMA compared to TMAO, leads to several beneficial metabolic outcomes^21–24^. Consistent with our observations, *Fmo3* deletion prevented hyperglycaemia, hyperlipidemia and atherosclerosis in mice and is considered a target for dysmetabolism^23^. Such a protective role for FMO3 inactivation cannot be explained by TMAO removal alone and therefore points to an independent mechanism for TMA.

Our discovery that TMA binds to and inhibits IRAK4, whereas TMAO does not, provides an unexpected and independent mechanism of action for TMA. Our observations on IRAK4 inhibition by TMA and in *Irak4* knockout mice are consistent with observations in Myd88 and *Irak1* knockout mice, resulting in improved glucose tolerance and insulin sensitivity. Collectively, we submit that our study reveals a context-dependent and target-specific mechanism of action independently of the proatherothrombic mechanism reported for TMAO in an HFD context. Modifying the TMA/TMAO ratio, for example, through FMO3 inhibition, would remove the deleterious proatherothrombic effect reported for TMAO and, through increased TMA concentration, would also blunt low-grade inflammation and improve glucose homoeostasis, in accordance with both mechanisms.

Our study is not without limitations. Notably, the derived IC_50_ from the purified kinase activity assay was approximately 250-fold higher than the *K*_d_ derived from the kinase screen. This may be a result of (1) the different properties reported upon by the different assay (physical binding for *K*_d_, activity for half-maximal inhibition for IC_50_); (2) the experimental design of the assays (IRAK4 bound to a drug-ligand versus ATP binding); (3) different kinase expression systems (bacterial *E.* *coli* versus insect Sf9 cells) and therefore potentially diverging IRAK4 catalytic activities (see Methods). Furthermore, the kinase screen tested 456 kinases out of the 518 kinases identified in the human kinome^12^, leaving the possibility that one of the 62 unscreened kinases could be indirectly involved, regardless of our focus on IRAK4-mediated LPS-signalling and HFD-signalling, which is consistent with established literature. Additionally, for the IRAK4 activity kinase assay, we used a relatively low ATP concentration (50 µM), which is orders of magnitude lower than the concentration in cells. Moreover, we did not calculate *K*_i_ at varying ATP concentrations. Instead, we used an IRAK4-ATP *K*_m_ derived from the literature (Methods), which may have affected the accuracy of the *K*_i_ that we report (0.7 µM). Using more stringent conditions (*K*_i_ of 3 µM, ATP *K*_m_ of 500 mM and assuming a cellular ATP concentration of 1–5 mM) would result in a cellular TMA IC_50_ for IRAK4 in the range of 9–30 µM, at the high end of physiological concentrations for TMA. We previously demonstrated in animals that plasma TMA concentrations can dramatically increase after a meal^19^, suggesting that TMA effects could be observed at the postprandial phase. Together with our findings here, this highlights how different meals rich in TMA precursors can affect plasma TMA levels, which will be tested in a human dietary intervention study. Despite the intrinsic limitations of the kinase assays, our work highlights the pathophysiological relevance of IRAK4 inhibition by TMA in two distinct cellular models (primary human hepatocytes and PBMCs) and four in vivo mouse models (LPS septic shock, HC-HFD, TMA supplementation and *Irak4* knockout) with TMA concentrations comparable to those found in human plasma, making these results biologically meaningful.

In conclusion, through the combination of ex vivo, in vitro and in vivo approaches, we reveal a unique mechanism in which TMA acts as a gut microbial signalling metabolite inhibiting IRAK4, a central molecular target in the TLR pathway, thereby allowing the gut microbiota to control HFD-induced pro-inflammatory response(s) and IR. The kinome represents a key repertoire of regulatory host targets for microbial signals, and the uncharted microbiome–kinome crosstalk requires further investigation. Our work can guide human trials in which the efficacy of IRAK4 inhibitors available for human use or dietary interventions that increase TMA bioavailability can be evaluated in the context of obesity and IR. Moreover, given that IR is an independent risk factor of cardiovascular disease in humans^72^, our work uncovers a strategy to alleviate obesity-associated increased cardiovascular risk. By highlighting the physiological and therapeutic roles of TMA and IRAK4 on HFD-induced low-grade inflammation and IR, we anticipate that its immunomodulatory properties extend past IR to a wider range of human pathologies involving TLR signalling and modulation of innate immunity.

## Methods

### Protocols

All experimental procedures involving mice were carried out in accordance with UK Home Office, Canadian Council on Animal Care, the Ethics Committee of the French Research Ministry (authorization number 00486.01), Belgian Law of May 29, 2013 regarding the protection of laboratory animals (agreement number LA1230314) and local guidelines on animal welfare and license conditions and the University of Oxford, University of Ottawa, Université Pierre et Marie Curie and Université catholique de Louvain guidelines on animal welfare.

### PBMC isolation and cell-based assays

PBMCs were isolated by our established method^73^, following local ethical approval from Imperial College Research Ethics Committee (19IC5372). Human peripheral blood samples (32 ml) were collected from donors in BD Vacutainer Cell Preparation Tubes containing Sodium Heparin/Ficoll (BD Biosciences). PBMCs were separated by centrifugation at 1600*g* for 30 min at room temperature (20–22 °C), followed by three washing steps with PBS and 10% FBS (LabTech) and centrifuged at 520 RCF for 10 min after each wash. Viability was measured using the Alamar Blue assay. Donors were females, aged between 25 and 32 years. All subjects were healthy volunteers by self-declaration and provided written informed consent.

### LPS stimulation and IL-6 and TNF release quantification

Human PBMCs (1 × 10^5^ per condition in duplicate), freshly isolated as described above, were serum-starved in 0.1% v/v FCS RPMI-1640 (Sigma-Aldrich, R7638) in the presence of TMA (Sigma-Aldrich, 72761) or the IRAK4 inhibitor PF06650833 (0.5 μM; Tocris, 6373) for 30 min, as indicated in the corresponding figures. PBMCs were subsequently stimulated with 1 μg ml^−1^ LPS (Sigma-Aldrich, L2630) for 4 h (37 °C, 5% CO_2_). For the phorbol 12-myristate 13-acetate (PMA)/ionomycin stimulation experiments, human PBMCs isolated as above (1 × 10^5^ per condition) pre-incubated with TMAO (100 μM), PF06650833 (0.5 μM) or vehicle as indicated for 30 min were then stimulated with 50 ng ml^−1^ PMA (Sigma-Aldrich, 79346) and 1 μg ml^−1^ ionomycin (Sigma-Aldrich, I0634) for 4 h. Next, cells were pelleted by centrifugation (200*g* for 10 min), and supernatants were collected and stored at −20 °C until further use. IL-6 and TNF were measured in the media diluted 1:10 in RPMI-1640 using the DuoSet ELISA kits (R&D, DY206 and DY210, respectively) according to the manufacturer’s instructions. For PMA/ionomycin TNF measurements, the media were undiluted. For all treatments, *n* = 4 biological repeats.

### PBMCs IRAK1 and NF-κBp65 phosphorylation in response to LPS

Freshly isolated human PBMCs (1 × 10^6^ per ml per condition) were added to 96-well plates coated with L-poly-lysine for 30 min (100 ng ml^−1^; Sigma-Aldrich, P4707) in RPMI-1640 containing 20% FCS and were left to attach to the wells overnight at 37 °C, 5% CO_2_. The following day, media were discarded and cells were incubated in serum-free RPMI-1640 containing 100 μM TMA, or RPMI-1640 vehicle for 30 min. PBMCs were then stimulated with LPS (1 μg ml^−1^) for up to 60 min (times indicated in the corresponding figure legends). At the end of the stimulation, PBMCs were fixed with 8% paraformaldehyde (Sigma-Aldrich, F8775) at room temperature for 20 min. Phosphorylation of IRAK1 (at Thr209) or NF-κBp65 (at Ser536) was determined in fixed PBMCs using commercially available ELISA kits (LSBio, LS-F1401-1 or LS-F891-1, respectively) according to the manufacturer’s instructions. Readings were normalized to the levels of total IRAK1 protein determined in sister wells, which were treated identically. The dose-dependent impact of TMA (see corresponding figures) on phosphorylation levels of IRAK1 or NF-κBp65 upon LPS (1 µg ml^−1^) stimulation of PBMCs (for 10 min or 15 min, respectively) was also determined using the commercially available kits from LSBio as described above.

### Cell-based assays in primary human hepatocytes

Cryopreserved primary human hepatocytes were commercially sourced (Innoprot) and cultured with hepatocytes medium (Innoprot) supplemented with 5% FBS, 1% hepatocytes growth supplement (mixture of growth factors, hormones and proteins necessary for culture of primary hepatocytes) and 100 U ml^−1^ penicillin and streptomycin. Human hepatocytes were grown on poly-L-lysine pre-coated cell dishes at 37 °C and a 5% CO_2_ atmosphere following the manufacturer’s recommendations. The following experiments were performed 24 h after seeding: first, palmitic acid (200 μM) administration (0 and 60 min) with or without TMA (0.1 mM, 30 min) pre-treatment, plus co-administration during palmitic acid exposure were assayed. The palmitic acid solution was prepared as previously reported^44,74^. In this experiment, the effect of palmitic acid and TMA on IRAK1, IRAK4, NF-κBp65, IKKαβ, SAPK/JNK and p38MAPK activity was analysed. In a second experiment, the effect of TMA (0.1 mM, 30 min) pre-treatment plus co-administration during vehicle or palmitic acid exposure for 4 h on insulin action and on IL-6 release was tested. Insulin action was evaluated by measuring p^Ser473^Akt1/Akt1 after insulin (100 nM, 10 min) stimuli.

p^Thr209^IRAK1, total IRAK1, p^Ser536^NF-κBp65, total NF-κBp65, p^Ser473^AKT1, total AKT1 and GAPDH (as an endogenous control) were measured with specific colourimetric cell-based ELISA Kits (Assay Biotechnology Company, CytoGlow IRAK1 (CBP1425), CytoGlow NF-κBp65 (CBP1633) and CytoGlow AKT1 (CBP1490), following the manufacturer’s instructions. For data analysis, the optical density of target proteins (read at 450 nm) was normalized with cell nuclei crystal violet staining (read at 595 nm), which was proportional to cell counts. The analysis of cell-based assays was performed in a blind manner.

p^Thr345/Ser346^IRAK4/IRAK4, p^Ser176/180^IKKαβ/IKKβ, p^Thr183/Tyr185^SAPK/JNK and p^Thr180/Tyr182^p38MAPK/p38MAPK were determined by western blot. In brief, hepatocyte proteins were directly extracted in radioimmunoprecipitation assay (RIPA) buffer (0.1% SDS, 0.5% sodium deoxycholate, 1% Nonidet P-40, 150 mM NaCl and 50 mM Tris-HCl pH 8.0) supplemented with protease inhibitors (1 mM phenylmethylsulfonyl fluoride). Cellular debris and lipids were eliminated by centrifugation of the solubilized samples at 13,000 rpm for 10 min at 4 °C, recovering the soluble fraction. Protein concentration was determined using the RC/DC Protein Assay (Bio-Rad Laboratories). RIPA protein extracts (20 μg) were separated by SDS–PAGE and transferred to nitrocellulose membranes by conventional procedures. Membranes were immunoblotted with antibodies against the following proteins: p^Thr345/Ser346^IRAK4 (11927), IRAK4 (4363), p^Ser176/180^IKKαβ (2694), IKKβ (8943), p^Thr183/Tyr185^SAPK/JNK (4668), SAPK/JNK (9258), p^Thr180/Tyr182^p38MAPK (9215) and p38MAPK (9212), all purchased from Cell Signaling Technology, and β-actin (sc-47778, Santa Cruz Biotechnology). Anti-rabbit IgG and anti-mouse IgG coupled to horseradish peroxidase was used as a secondary antibody. Horseradish peroxidase activity was detected by chemiluminescence, and quantification of protein expression was performed using Scion Image software. IL-6 concentration in hepatocyte media was measured using Human IL-6 Quantikine ELISA Kit (R&D Systems, D6050). All experiments were performed with at least three sample replicates.

### Mouse models

#### Longitudinal HFD feeding in mice

All experiments were approved by the ethical committee of the University of Oxford. Male mice from a C57BL/6J inbred strain were bred in our animal facility by using a stock originating from The Jackson Laboratory. At 5 weeks of age, groups of eight to ten mice were transferred to a 40% w/w HFD (65% kcal) (Special Diets Services, 824155b), containing 32% lard and 8% corn oil, whereas control groups remained on a normal carbohydrate (CHD) diet containing 5% fat, 19% protein and 3.5% fibre (B&K rat and mouse pelleted diet, B&K Universal) for up to 6 months. Detailed diet formulations were published previously^13^ and are summarized in Supplementary Table 6.

Mice were housed under a 12 h–12 h light–dark cycle. For physiological profiling, several mouse groups fed CHD or HFD were tested to assess the consistency of results and discard any impact of potential batch effects. Intraperitoneal GTTs were performed on 2, 3, 5 and 7-month-old mice after an overnight fast, as previously published^75^ (see also metabolic phenotyping below). Then, 4 days after the GTT, 24 h urinary samples (09:00–21:00 h) were collected from mice maintained in individual metabolic cages. Urinary samples collected in a solution of 1% (wt/vol) sodium azide were centrifuged to remove solid particles and kept at −80 °C until assayed. After an overnight fast, mice were killed by exsanguination. Plasma was separated by centrifugation and stored at −80 °C until ^1^H-NMR analysis.

#### Choline supplementation on HFD

At 5 weeks of age, mice were fed either a CHD containing 2 g of choline per kg of diet (Research Diets, D12450J), a LC-HFD containing 2 g of choline per kg of diet (Research Diets, D12492), a HC-HFD containing 17 g of choline per kg of diet (Research Diets, D16100401), a HC-HFD containing 1% of DMB or a HC-HFD combined with a cocktail of antibiotics (0.5 g l^−1^ vancomycin hydrochloride, 1 g l^−1^ neomycin trisulfate, 1 g l^−1^ metronidazole, 1 g l^−1^ ampicillin sodium) in drinking bottles (*n* = 6–10 per group) for 8 weeks (see diet formulations in Supplementary Table 6). Mice were then killed by decapitation, and organs were dissected and weighed.

#### *Irak4*^−/−^ mice

*Irak4*^−/−^ mice on a C57BL/6J background, as previously described^38,45^, were bred with C57BL/6J mice (The Jackson Laboratory), and the F1 offspring were subsequently bred to produce the *Irak4*^−/−^ mice and wild-type littermates used for this study. Mice were bred and genotyped at the Animal Facility of the University of Ottawa Heart Institute. The following primers were used for genotyping. *Irak4* knockout, 5′-TGAATGGAAGGATTGGAGCTACGGGGGT-3′; *Irak4* common, 5′-GAACACGCTCCCAGGTCTCTTTCCAAC-3′; and *Irak4* wild-type, 5′-TCTTCTACCTGAAATATGAAAGATTCCT-3′. The PCR reaction was run at 94 °C for 60 s, 60 °C for 60 s and 72 °C for 60 s for 40 cycles. The mice (10–12 weeks old) were fed with HFD for 8 weeks and then killed by decapitation, and their organs were dissected and weighed at the end of the study.

#### Chronic TMA and PF06650833 treatment in LC-HFD-fed mice

C57BL/6J mice (Charles River; 5 weeks old) were housed for 1 week before the experiment in a controlled environment. Mice were maintained under a 12 h–12 h light–dark cycle. On day 0, the 10-week-old mice were anaesthetized with isoflurane (ForeneH, Abbott). Mini-osmotic pumps were implanted subcutaneously (Model 2006, Alzet) (flow rate, 0.15 ml h^−1^; total filling volume, 200 ml; delivery duration, 42 days) as previously described^76^. The osmotic mini-pump contained either vehicle or TMA (0.1 mM in circulation) or PF06650833 (50 nM in circulation). After 6 weeks of metabolite treatment, mice were killed by decapitation, and the organs were dissected and weighed.

#### Septic shock mouse model

Male mice on a C57BL/6J background (6 weeks old) were purchased from Charles River and maintained in a controlled environment for 2 weeks to acclimate them to local conditions. The animal experimental protocol was approved by local and national committees in charge (Tor Vergata University Institutional Animal Care and Use Committee and Ministry of Health, license no. 265/2019-PR) and conducted in accordance with accepted standards of humane animal care. Mice were intraperitoneally injected with 59 mg kg^−1^ TMA (Sigma-Aldrich, 72761) (treatment group, *n* = 7) or PBS alone (control group, *n* = 6) 30 min before LPS injection (30 mg kg^−1^ of LPS (Sigma-Aldrich, L2630) in sterile PBS by intraperitoneal injection). The survival of the mice was monitored every 4 h for 36 h.

Mice were housed in standard ventilated cages under a 12 h–12 h light–dark cycle at 20–23 °C and 40–60% humidity. For mouse experiments, staff who were responsible for phenotyping and biospecimen collection also handled the dietary intervention; therefore, they were not blinded to experimental conditions.

### Physiological phenotyping

After 4 weeks of treatment, an intraperitoneal GTT test (2 g kg^−1^) was performed in conscious mice following an overnight fast. Blood was collected from the tail vein before glucose injection and 30, 60, 90 and 120 min afterwards. Blood glucose levels were determined using an Accu-Check Performa (Roche Diagnostics). Additional blood samples were collected at baseline and 30 min after glucose injection in Microvette CB 300 Lithium Heparin (Sarstedt). Plasma was separated by centrifugation and stored at −80 °C until the insulin radioimmunoassay. Circulating insulin levels were determined using insulin ELISA kits (Mercodia). The Matsuda insulin sensitivity index was calculated as previously published^31^.

After 5 weeks, we performed an ITT. Mice that had been fasted for 5 h were injected intraperitoneally with insulin (0.75 mU g^−1^; Actrapid, Novo Nordisk). Blood glucose levels were measured immediately before and 15, 30, 45, 60, 90 and 120 min after insulin injection with a standard glucose meter (Accu-Check, Roche) on the tip of the tail vein.

### Gene expression

Groups of six mice showing consistent pathophysiological profiles in response to CHD or HFD treatment were selected for microarray analysis performed with our established method^75^, and data were deposited in ArrayExpress under accession number E-MEXP-1755. Total RNA was isolated from frozen liver tissue using the Trizol reagent (Invitrogen Life Technologies), followed by further purification with RNeasy spin columns (Qiagen). The concentration and quality of RNA samples were evaluated using an Agilent 2100 Bioanalyser (Agilent Technologies). Gene expression analysis was performed using the Affymetrix Mouse Genome arrays (U430A and U430B). These arrays include 22,690 (U430A) and 22,576 (U430B) probe sets, enabling the detection of transcript levels for approximately 13,250 (U430A) and 7,577 (U430B) unique genes and expressed sequence tags. For each sample, 10 μg of total RNA was used for first-strand cDNA synthesis, followed by in vitro transcription to generate biotin-labelled complementary RNA (cRNA). The cRNA samples were then assessed for yield and integrity using an Agilent 2100 Bioanalyser. After fragmentation, individual cRNA preparations were hybridized to the microarrays using a temperature-controlled Affymetrix hybridization oven. Post-hybridization washing and staining were carried out using the Affymetrix Fluidics Station 450. Finally, the arrays were scanned at a wavelength of 560 nm with an Agilent scanner (Affymetrix). The Bioconductor^77^ package Limma^78^ was used to generate the list of differentially expressed genes. Gene ontology was implemented using Enrichr^79^, and signalling pathway impact analysis was conducted using SPIA^80^.

For qPCR analysis, total RNA was prepared from tissues using TriPure reagent (Roche). Quantification and integrity analysis of total RNA were performed by analysing 1 µl of each sample in an Agilent 2100 Bioanalyzer (Agilent RNA 6000 Nano Kit). cDNA was prepared by reverse transcription of 1 mg total RNA using a Reverse Transcription System kit (Promega). Real-time PCR was performed with the StepOnePlus real-time PCR system and software (Applied Biosystems) using Mesa Fast qPCR (Eurogentec) for detection according to the manufacturer’s instructions. RPL19 RNA was chosen as the housekeeping gene. All samples were performed in duplicate in a single 96-well reaction plate, and data were analysed according to the 2^−ΔΔCT^ method. The identity and purity of the amplified product were assessed by melting curve analysis at the end of amplification. The primer sequences for the targeted mouse genes are as follows: SAA1 forward, CATTTGTTCACGAGGCTTTCC; SAA1 reverse, GTTTTTCCAGTTAGCTTCCTTCATGT; SAA2 forward, GGGGTCTGGGCTTCCCATCT; SAA2 reverse, CCATTCTGAAACCCTTGTGG; SAA3 forward, CGCAGCACGAGCAGGAT; SAA3 reverse, CCAGGATCAAGATGCAAAGAATG as previously reported^81^. Quantitative PCR with reverse transcription assays were performed in a single batch, with the personnel blinded to treatment groups.

### Circulating cytokine quantification

Circulating cytokines were quantified using the MSD V-PLEX Plus Proinflammatory Panel 1 kit. Plasma samples were diluted two times in the diluent provided, and the experiment was processed as outlined by the manufacturer and read on a SECTOR imager 2400. Cytokine assays were performed in a single batch with the personnel blinded to treatment groups.

### Western blotting

Western blot analyses were performed according to our established methods^82^. To analyse the insulin signalling pathway, mice were allocated to either a saline-injected subgroup or an insulin-injected subgroup so that both subgroups were matched in terms of body weight and fat mass. They then received 1 mU insulin per g body weight (Actrapid; Novo Nordisk) or an equal volume of saline solution. Then, 3 min after injection, the mice were killed and their liver was dissected. A total of 30 mg of liver was homogenized in 680 µl of RIPA buffer containing a cocktail of protease and phosphatase inhibitors. The homogenate was then centrifuged at 12,000*g* for 20 min at 4 °C. Equal amounts of proteins were separated by SDS–PAGE and transferred to nitrocellulose membranes. Membranes were incubated overnight at 4 °C with antibodies diluted in Tris-buffered saline Tween-20 containing 1% BSA:p-Akt Ser473 (1:1,000; Cell Signaling Technology, 4060), total Akt (1:1,000; Cell Signalling Technology, 9272S), p-NF-κB (1:3,000; AbCam, ab86299) and total NF-κB p65 (1:3,000; Cell Signalling Technology, 8242). Insulin-induced p-Akt/total Akt corresponds to the ratio between p-Akt/Akt in insulin-treated mice and p-Akt/Akt in saline-treated mice. For these western blots, the membranes were stripped and re-probed with a β-actin antibody as a loading control. In separate densitometric analyses, we additionally corrected total Akt levels with β-actin and subsequently used this ratio to correct p-Akt, so as to take into account total protein levels.

### ^1^H-NMR spectroscopy and multivariate statistics

Mouse urine samples were prepared by mixing 200 μl of urine with 200 μl of distilled water and 200 μl of 0.1 M phosphate buffer (containing 10% D_2_O/H_2_O, v/v, and 0.05% sodium 3-trimethylsilyl-(2,2,3,3-^2^H_4_)-1-propionate as a chemical shift reference at δ 0.0). The mixtures were loaded into 96-well plates for high-throughput flow-injection NMR spectroscopy. Samples were prepared and measured on a spectrometer (Bruker) operating at 600.22 MHz ^1^H frequency as detailed previously^13^. In short, a standard 1D pulse sequence (recycle delay-90°-*t*_1_-90°-*t*_m_-90°-acquisition) was used. Water suppression was performed by irradiating the water peak during the recycle delay (2 s) and mixing time, *t*_m_ (150 ms); *t*_1_ was set to 3 μs. The 90° pulse length was adjusted to ≈10 μs. We acquired 128 transients at 32,000 a data point resolution for each spectrum with a 20 ppm spectral width. Free induction decays were multiplied by an exponential function corresponding to a 0.3-Hz line-broadening factor before Fourier transformation. The NMR spectra were corrected for phase and baseline. Full-resolution ^1^H-NMR spectra were imported, and the area corresponding to the water region after water suppression (δ4.5–5.0) was discarded. The full-resolution spectra were then processed and analysed using O-PLS-DA as we had done previously^13^. In this version of discriminant analysis, class separation is maximized by using NMR data (*X*) to model the class matrix (*Y*, with *n* dummy variables for *n* classes), through decomposition of the covariance matrix (*Y*ᵀ*X*) into *n* − 1 O-PLS components and additional orthogonal signal correction components^13^. Variance component analysis was performed as described previously^83^. ^1^H-NMR profiling was performed in a single batch with the personnel blinded to treatment groups. The O-PLS-DA model was validated using 10,000 random permutations of the original class membership variable to explain (that is, diets, treatments or genotypes), as described previously^83^.

### Plasma methylamine quantification by UPLC–MS/MS

Methylamines were quantified according to our previously validated methods^29,30^: plasma samples (20 μl) were spiked with 10 μl internal standard solution (^13^C_3_/^15^N-TMA, d_9_-TMAO, d_4_-choline, d_3_-carnitine and d_9_-betaine in water; 1 mg l^−1^) and 45 μl of ethyl 2-bromoacetate solution (15 g l^−1^ ethyl 2-bromoacetate, 1% NH_4_OH in acetonitrile) were added to derivatize methylamines (TMA and ^13^C_3_/^15^N-TMA) to their ethoxy-analogues, completed after 30 min at room temperature. Methylamines were derivatised with ethyl bromoacetate to increase sensitivity (the underivatized form elicited a low response from the mass spectrometer owing to its low molecular weight) and enhance chromatographic performance. A total of 935 μl of a protein/lipid precipitation solution (94% acetonitrile/5% water/1% formic acid) was added; samples were centrifuged for 20 min (4 °C, 20,000*g*) and transferred to UPLC–autosampler vials. Sample injections (10 μl loop) were performed on a Waters Acquity UPLC-Xevo TQ-S UPLC–MS/MS system equipped with an Acquity BEH HILIC (2.1 × 100 mm, 1.7 μm) chromatographic column. An isocratic elution was applied with 10 mM ammonium formate in 95:5 (v/v) acetronitrile:water for 14 min at 500 μl min^−1^ and 50 °C. Positive electrospray (ESI+) was used as the ionization source, and mass spectrometer parameters were set as follows: capillary, cone and source offset voltages at 500, 93 and 50 V, respectively; desolvation temperature at 600 °C; desolvation/cone/nebulizer gases were high-purity nitrogen at 1,000 l h^−1^, 150 l h^−1^ and 7 bar, respectively. Collision gas was high-purity argon. The mass spectrometer was operated in multiple reaction monitoring mode. The monitored transitions were as follows: for derivatized-TMA, +146 → +118/59 *m*/*z* (23/27 V); for derivatised-^13^C_3_/^15^N-TMA, +150 → +63 (27 V); for TMAO, +76 → +59/58 *m*/*z* (12/13 V); for d_9_-TMAO, +85 → +68 *m*/*z* (18 V); for choline, +104 → +60/45 *m*/*z* (14/16 V); for d_4_-choline, +108 → +60 *m*/*z* (15 V); for γ-butyrobetaine, +146 → +60/87 *m*/*z* (12/12 V); for carnitine, +162 → +103/60 *m*/*z* (16/14 V); for d_3_-carnitine, +165 → +103 *m*/*z* (16 V); for betaine, +118 → +59/58 *m*/*z* (16/16 V); and for d_9_-betaine, +127 → +68 *m*/*z* (16 V).

### Kinome screen, *K*_*d*_

TMA was assessed using the KdELECT screening service (DiscoveRx) as described previously^35,36^. This technique is based on a competition-binding assay that quantitatively measures the ability of a compound to compete with an immobilized, active-site-directed ligand. The assay consists of a DNA-tagged kinase, an immobilized ligand and the potent inhibitor. The ability of TMA to compete with the immobilized ligand was measured by qPCR of the DNA tag. *K*_*d*_ was then calculated from a duplicate 11-point dose–response curve. Kinase interaction tree plots were generated using the TREEspot Software Tool and are reprinted with permission from KINOMEscan, a division of DiscoveRx Corporation, 2015.

### Kinase activity assays

The TMA IC_50_ on IRAK4 was determined using Kinexus kinase-inhibitor activity profiling service (Kinexus). Protein kinase assays (in duplicate) were performed at ambient temperature for 30 min in a final volume of 25 μl according to the following assay reaction recipe:

#### Component 1

A total of 5 μl of diluted active IRAK4 target (recombinant, full length, expressed by baculovirus in Sf9 insect cells with an GST tag (SignalChem Catalogue 112-10G); ~10–50 nM final concentration in the assay).

#### Component 2

A total of 5 μl of stock solution of substrate (Myelin Basic Protein 1 mg ml^−1^ diluted in H_2_O).

#### Component 3

A total of 5 μl of kinase assay buffer (25 mM MOPS pH 7.2, 12.5 mM β-glycerol-phosphate, 25 mM MgCl_2_, 5 mM EGTA, 2 mM EDTA, 0.25 mM dithiothreitol, added just before assay initiation).

#### Component 4

A total of 5 μl of compound (various concentrations as indicated) or 10% dimethylsulfoxide for blank.

#### Component 5

A total of 5 μl of ^32^P-ATP (250 μM stock solution, 0.8 μCi, Perkin Elmer).

The assay was initiated by the addition of ^32^P-ATP, and the reaction mixture was incubated at ambient temperature for 30 min. After the incubation period, the assay was terminated by spotting 10 μl of the reaction mixture onto a Multiscreen phosphocellulose P81 plate, which was washed three times, each time for approximately 15 min, in a 1% phosphoric acid solution. The radioactivity on the P81 plate was counted in the presence of scintillation fluid in a Trilux scintillation counter. Blank control was set up that included all the assay components except the addition of Myelin Basic Protein (replaced with an equal volume of assay dilution buffer). The corrected activity for the IRAK4 target was determined by removing the blank control value.

We calculated the TMA *K*_i_ for IRAK4 using the equation below^84^, where IC_50_ = 3.4 µM, as determined from the IRAK4 activity assay described above. [S] is the concentration of ATP in the assay (50 µM), and the *K*_m_ value of IRAK4 for ATP (*K*_m_ = 13.6 µM) was provided by the commercial vendor. Purified IRAK4 for these assays was obtained from https://media.cellsignal.com/pdf/7551.pdf.

$${K}_{{\rm{i}}}=\frac{{\mathrm{IC}}_{50}}{1+\frac{[{\boldsymbol{S}}]}{{K}_{{\rm{m}}}}}$$

### Reagents

Glutamine (Glutamax, 35050061, Life Technologies), FBS (Life Technologies), crystal violet (C6158) and trimethylamine solution (W324108) were obtained from Sigma-Aldrich. Mouse IL-6 Quantikine ELISA kits (M6000B) were obtained from R&D Systems, and the RNeasy Micro Kit was from Qiagen. SuperScript II Reverse Transcriptase, IL-6 Taqman probe Hs00174131_m1 and FAST master mix were purchased from Invitrogen. The HFD (Special Diets Services) and CHD (B&K Universal) were specifically formulated as described in a previous publication^12^; the remaining foods were obtained from Research Diets: control diet (D12450K), LC-HFD (60% kcal fat and 20% kcal carbohydrates; D12492), and HC-HFD (60% kcal fat and 20% kcal carbohydrates with 17 g of choline per kg; D16100401i). The mouse insulin ELISA was obtained from Mercodia (10-1249-01). Additional reagents and kits included isoflurane (10014451, Forene, Abbott); TriPure reagent (1667165, Roche); Reverse Transcription System kit (A3500, Promega); Mesa Fast qPCR (CS-CKIT-PROD, Eurogentec); and the MSD V-PLEX Plus Proinflammatory Panel 1 kit (K15048G Meso Scale Diagnostics).

### Statistics

Potential outliers were identified by a Grubbs test. For statistical comparisons between study groups, normality was tested using the D’agostino–Pearson omnibus normality test, then one-way ANOVA was used, followed by Tukey’s post hoc testing when data were normally distributed; otherwise, groups were compared using the two-tailed Mann–Whitney test (*P* < 0.05 considered to be statistically significant). Where applicable, *P* values were corrected for multiple comparisons using the Benjamini–Hochberg method, unless otherwise stated. Data are displayed as means ± s.e.m in all figures. All cell culture experiments included at least three biological replicates (as indicated in figure legends). All animal cohorts included at least five animals in each study group (as indicated in figure legends); animals were randomized to treatment groups and were sampled in a random order. Data collection and analysis were not performed blind to the conditions of the experiments. No statistical methods were used to pre-determine sample sizes, but our sample sizes are similar to those reported in our previous publications^69^.

### Reporting summary

Further information on research design is available in the Nature Portfolio Reporting Summary linked to this article.

## Supplementary information

Reporting Summary

Supplementary Tables Supplementary Tables 1–6.

## Source data

Source Data Fig. 1 Statistical Source Data and/or gels.

Source Data Fig. 2 Statistical Source Data and/or gels.

Source Data Fig. 3 Statistical Source Data and/or gels.

Source Data Fig. 4 Statistical Source Data and/or gels.

Source Data Fig. 5 Statistical Source Data and/or gels.

Source Data Extended Data Fig. 1 Statistical Source Data and/or gels.

Source Data Extended Data Fig. 2 Statistical Source Data and/or gels.

Source Data Extended Data Fig. 3 Statistical Source Data and/or gels.

Source Data Extended Data Fig. 4 Statistical Source Data and/or gels.

Source Data Extended Data Fig. 5 Statistical Source Data and/or gels.

Source Data Extended Data Fig. 6 Statistical Source Data and/or gels.

Source Data Extended Data Fig. 7 Statistical Source Data and/or gels.

## Data Availability

Microarray data are deposited in ArrayExpress under accession number E-MEXP-1755. ^1^H-NMR-based metabolomics data are deposited in Metabolights with accession number MTBLS12989 (https://www.ebi.ac.uk/metabolights/MTBLS12989). The UPLC–MS/MS spectra for isotopically quantified methylamines are deposited in Metabolights with accession number MTBLS12975 (https://www.ebi.ac.uk/metabolights/MTBLS12975). Source data are provided with this paper.

## Code availability

No custom code or algorithm was used for the analyses conducted in this work.

## References

[1] Ong, K. L. et al. Global, regional, and national burden of diabetes from 1990 to 2021, with projections of prevalence to 2050: a systematic analysis for the Global Burden of Disease Study 2021. *Lancet* **402**, 203–234 (2023). 37356446 10.1016/S0140-6736(23)01301-6 PMC10364581

[2] Samuel, V. T. & Shulman, G. I. Mechanisms for insulin resistance: common threads and missing links. *Cell* **148**, 852–871 (2012). 22385956 10.1016/j.cell.2012.02.017 PMC3294420

[3] Cai, D. et al. Local and systemic insulin resistance resulting from hepatic activation of IKK-β and NF-κB. *Nat. Med.* **11**, 183–190 (2005). 15685173 10.1038/nm1166 PMC1440292

[4] Wu, G. D. et al. Linking long-term dietary patterns with gut microbial enterotypes. *Science* **334**, 105–108 (2011). 21885731 10.1126/science.1208344 PMC3368382

[5] Le Chatelier, E. et al. Richness of human gut microbiome correlates with metabolic markers. *Nature* **500**, 541–546 (2013). 23985870 10.1038/nature12506

[6] Pedersen, H. K. et al. Human gut microbes impact host serum metabolome and insulin sensitivity. *Nature* **535**, 376–381 (2016). 27409811 10.1038/nature18646

[7] Cani, P. D. et al. Metabolic endotoxemia initiates obesity and insulin resistance. *Diabetes* **56**, 1761–1772 (2007). 17456850 10.2337/db06-1491

[8] Hooper, L. V., Littman, D. R. & Macpherson, A. J. Interactions between the microbiota and the immune system. *Science* **336**, 1268–1273 (2012). 22674334 10.1126/science.1223490 PMC4420145

[9] Nicholson, J. K. et al. Host–gut microbiota metabolic interactions. *Science* **336**, 1262–1267 (2012). 22674330 10.1126/science.1223813

[10] Maslowski, K. M. et al. Regulation of inflammatory responses by gut microbiota and chemoattractant receptor GPR43. *Nature* **461**, 1282–1286 (2009). 19865172 10.1038/nature08530 PMC3256734

[11] Venkatesh, M. et al. Symbiotic bacterial metabolites regulate gastrointestinal barrier function via the xenobiotic sensor PXR and Toll-like receptor 4. *Immunity* **41**, 296–310 (2014). 25065623 10.1016/j.immuni.2014.06.014 PMC4142105

[12] Manning, G., Whyte, D. B., Martinez, R., Hunter, T. & Sudarsanam, S. The protein kinase complement of the human genome. *Science* **298**, 1912–1934 (2002). 12471243 10.1126/science.1075762

[13] Dumas, M.-E. et al. Metabolic profiling reveals a contribution of gut microbiota to fatty liver phenotype in insulin-resistant mice. *Proc. Natl Acad. Sci. USA* **103**, 12511–12516 (2006). 16895997 10.1073/pnas.0601056103 PMC1567909

[14] Wang, Z. et al. Gut flora metabolism of phosphatidylcholine promotes cardiovascular disease. *Nature* **472**, 57–63 (2011). 21475195 10.1038/nature09922 PMC3086762

[15] Al-Waiz, M., Mikov, M., Mitchell, S. C. & Smith, R. L. The exogenous origin of trimethylamine in the mouse. *Metabolism* **41**, 135–136 (1992). 1736035 10.1016/0026-0495(92)90140-6

[16] Craciun, S. & Balskus, E. P. Microbial conversion of choline to trimethylamine requires a glycyl radical enzyme. *Proc. Natl Acad. Sci. USA* **109**, 21307–21312 (2012). 23151509 10.1073/pnas.1215689109 PMC3535645

[17] Koeth, R. A. et al. Intestinal microbiota metabolism of l-carnitine, a nutrient in red meat, promotes atherosclerosis. *Nat. Med.* **19**, 576–585 (2013). 23563705 10.1038/nm.3145 PMC3650111

[18] Al-Waiz, M., Ayesh, R., Mitchell, S. C., Idle, J. R. & Smith, R. L. Disclosure of the metabolic retroversion of trimethylamine *N*-oxide in humans: a pharmacogenetic approach. *Clin. Pharmacol. Ther.* **42**, 608–612 (1987). 3690938 10.1038/clpt.1987.207

[19] Hoyles, L. et al. Metabolic retroconversion of trimethylamine *N*-oxide and the gut microbiota. *Microbiome* **6**, 73 (2018). 29678198 10.1186/s40168-018-0461-0 PMC5909246

[20] Dolphin, C. T., Janmohamed, A., Smith, R. L., Shephard, E. A. & Phillips, L. R. Missense mutation in flavin-containing mono-oxygenase 3 gene, *FMO3*, underlies fish-odour syndrome. *Nat. Genet.* **17**, 491–494 (1997). 9398858 10.1038/ng1297-491

[21] Shih, D. M. et al. Flavin containing monooxygenase 3 exerts broad effects on glucose and lipid metabolism and atherosclerosis. *J. Lipid Res.* **56**, 22–37 (2015). 25378658 10.1194/jlr.M051680 PMC4274068

[22] Warrier, M. et al. The TMAO-generating enzyme flavin monooxygenase 3 is a central regulator of cholesterol balance. *Cell Rep.* **10**, 326–338 (2015). 25600868 10.1016/j.celrep.2014.12.036 PMC4501903

[23] Miao, J. et al. Flavin-containing monooxygenase 3 as a potential player in diabetes-associated atherosclerosis. *Nat. Commun.* **6**, 6498 (2015). 25849138 10.1038/ncomms7498 PMC4391288

[24] Schugar, R. C. et al. The TMAO-producing enzyme flavin-containing monooxygenase 3 regulates obesity and the beiging of white adipose tissue. *Cell Rep.* **19**, 2451–2461 (2017). 28636934 10.1016/j.celrep.2017.05.077 PMC5672822

[25] Özcan, U. et al. Endoplasmic reticulum stress links obesity, insulin action, and type 2 diabetes. *Science* **306**, 457–461 (2004). 15486293 10.1126/science.1103160

[26] Eckel-Mahan, K. L. et al. Reprogramming of the circadian clock by nutritional challenge. *Cell* **155**, 1464–1478 (2013). 24360271 10.1016/j.cell.2013.11.034 PMC4573395

[27] Thorens, B. et al. Use of preclinical models to identify markers of type 2 diabetes susceptibility and novel regulators of insulin secretion—a step towards precision medicine. *Mol. Metab.* **27**, S147–S154 (2019). 10.1016/j.molmet.2019.06.008 PMC6768503 31500826

[28] Dumas, M.-E. et al. Microbial-host co-metabolites are prodromal markers predicting phenotypic heterogeneity in behavior, obesity, and impaired glucose tolerance. *Cell Rep.* **20**, 136–148 (2017). 28683308 10.1016/j.celrep.2017.06.039 PMC5507771

[29] Fromentin, S. et al. Microbiome and metabolome features of the cardiometabolic disease spectrum. *Nat. Med.* **28**, 303–314 (2022). 35177860 10.1038/s41591-022-01688-4 PMC8863577

[30] Forslund, S. K. et al. Combinatorial, additive and dose-dependent drug–microbiome associations. *Nature* **600**, 500–505 (2021). 34880489 10.1038/s41586-021-04177-9

[31] Matsuda, M. & DeFronzo, R. A. Insulin sensitivity indices obtained from oral glucose tolerance testing: comparison with the euglycemic insulin clamp. *Diabetes Care* **22**, 1462–1470 (1999). 10480510 10.2337/diacare.22.9.1462

[32] Liu, H.-Y. et al. Increased basal level of Akt-dependent insulin signaling may be responsible for the development of insulin resistance. *Am. J. Physiol. Endocrinol. Metab.* **297**, E898–E906 (2009). 19638508 10.1152/ajpendo.00374.2009 PMC2763787

[33] Sack, G. H. Serum amyloid A—a review. *Mol. Med.* **24**, 46 (2018). 30165816 10.1186/s10020-018-0047-0 PMC6117975

[34] Wang, Z. et al. Non-lethal inhibition of gut microbial trimethylamine production for the treatment of atherosclerosis. *Cell* **163**, 1585–1595 (2015). 26687352 10.1016/j.cell.2015.11.055 PMC4871610

[35] Fabian, M. A. et al. A small molecule–kinase interaction map for clinical kinase inhibitors. *Nat. Biotechnol.* **23**, 329–336 (2005). 15711537 10.1038/nbt1068

[36] Karaman, M. W. et al. A quantitative analysis of kinase inhibitor selectivity. *Nat. Biotechnol.* **26**, 127–132 (2008). 18183025 10.1038/nbt1358

[37] Sun, X.-J. et al. Deletion of interleukin 1 receptor-associated kinase 1 (*Irak1*) improves glucose tolerance primarily by increasing insulin sensitivity in skeletal muscle. *J. Biol. Chem.* **292**, 12339–12350 (2017). 28572512 10.1074/jbc.M117.779108 PMC5519380

[38] Suzuki, N. et al. Severe impairment of interleukin-1 and Toll-like receptor signalling in mice lacking IRAK-4. *Nature* **416**, 750–754 (2002). 11923871 10.1038/nature736

[39] Picard, C. et al. Pyogenic bacterial infections in humans with IRAK-4 deficiency. *Science* **299**, 2076–2079 (2003). 12637671 10.1126/science.1081902

[40] Lee, K. L. et al. Discovery of clinical candidate 1-{[(2 *S*, 3 *S*, 4 *S*)-3-Ethyl-4-fluoro-5-oxopyrrolidin-2-yl]methoxy}-7-methoxyisoquinoline-6-carboxamide (PF-06650833), a potent, selective inhibitor of interleukin-1 receptor associated kinase 4 (IRAK4), by fragment-based drug design. *J. Med. Chem.* **60**, 5521–5542 (2017). 28498658 10.1021/acs.jmedchem.7b00231

[41] Ku, C.-L. et al. Selective predisposition to bacterial infections in IRAK-4–deficient children: IRAK-4-dependent TLRs are otherwise redundant in protective immunity. *J. Exp. Med.* **204**, 2407–2422 (2007). 17893200 10.1084/jem.20070628 PMC2118442

[42] Kollewe, C. et al. Sequential autophosphorylation steps in the interleukin-1 receptor-associated kinase-1 regulate its availability as an adapter in interleukin-1 signaling. *J. Biol. Chem.* **279**, 5227–5236 (2004). 14625308 10.1074/jbc.M309251200

[43] Kim, T. W. et al. A critical role for IRAK4 kinase activity in Toll-like receptor-mediated innate immunity. *J. Exp. Med.* **204**, 1025–1036 (2007). 17470642 10.1084/jem.20061825 PMC2118590

[44] Hoyles, L. et al. Molecular phenomics and metagenomics of hepatic steatosis in non-diabetic obese women. *Nat. Med.* **24**, 1070–1080 (2018). 29942096 10.1038/s41591-018-0061-3 PMC6140997

[45] Valaperti, A. et al. Innate immune interleukin-1 receptor-associated kinase 4 exacerbates viral myocarditis by reducing CCR5^+^ CD11b^+^ monocyte migration and impairing interferon production. *Circulation* **128**, 1542–1554 (2013). 24030499 10.1161/CIRCULATIONAHA.113.002275

[46] Winkler, A. et al. The interleukin-1 receptor-associated kinase 4 inhibitor PF-06650833 blocks inflammation in preclinical models of rheumatic disease and in humans enrolled in a randomized clinical trial. *Arthritis Rheumatol.* **73**, 2206–2218 (2021). 34423919 10.1002/art.41953 PMC8671219

[47] O’Neill, L. A. J., Golenbock, D. & Bowie, A. G. The history of Toll-like receptors—redefining innate immunity. *Nat. Rev. Immunol.* **13**, 453–460 (2013). 23681101 10.1038/nri3446

[48] von Bernuth, H., Picard, C., Puel, A. & Casanova, J.-L. Experimental and natural infections in MyD88- and IRAK-4-deficient mice and humans. *Eur. J. Immunol.* **42**, 3126–3135 (2012). 23255009 10.1002/eji.201242683 PMC3752658

[49] Caesar, R., Tremaroli, V., Kovatcheva-Datchary, P., Cani, P. D. & Bäckhed, F. Crosstalk between gut microbiota and dietary lipids aggravates WAT inflammation through TLR signaling. *Cell Metab.* **22**, 658–668 (2015). 26321659 10.1016/j.cmet.2015.07.026 PMC4598654

[50] Shi, H. et al. TLR4 links innate immunity and fatty acid–induced insulin resistance. *J. Clin. Invest.* **116**, 3015–3025 (2006). 17053832 10.1172/JCI28898 PMC1616196

[51] Reilly, S. M. et al. An inhibitor of the protein kinases TBK1 and IKK-ɛ improves obesity-related metabolic dysfunctions in mice. *Nat. Med.* **19**, 313–321 (2013). 23396211 10.1038/nm.3082 PMC3594079

[52] Kiechl, S. et al. Blockade of receptor activator of nuclear factor-κB (RANKL) signaling improves hepatic insulin resistance and prevents development of diabetes mellitus. *Nat. Med.* **19**, 358–363 (2013). 23396210 10.1038/nm.3084

[53] Bain, M. A., Faull, R., Fornasini, G., Milne, R. W. & Evans, A. M. Accumulation of trimethylamine and trimethylamine-*N*-oxide in end-stage renal disease patients undergoing haemodialysis. *Nephrol. Dial. Transplant.* **21**, 1300–1304 (2006). 16401621 10.1093/ndt/gfk056

[54] Lundh, T., Akesson, B. & Skerfving, S. Effect of dietary intake of trimethylamine on human metabolism of the industrial catalyst dimethylethylamine. *Occup. Environ. Med.* **52**, 478–483 (1995). 7670623 10.1136/oem.52.7.478 PMC1128267

[55] Dumas, M.-E. The microbial–mammalian metabolic axis: beyond simple metabolism. *Cell Metab.* **13**, 489–490 (2011). 21531329 10.1016/j.cmet.2011.04.005

[56] Spencer, M. D. et al. Association between composition of the human gastrointestinal microbiome and development of fatty liver with choline deficiency. *Gastroenterology* **140**, 976–986 (2011). 21129376 10.1053/j.gastro.2010.11.049 PMC3049827

[57] Gao, X., Wang, Y. & Sun, G. High dietary choline and betaine intake is associated with low insulin resistance in the Newfoundland population. *Nutrition* **33**, 28–34 (2017). 27908547 10.1016/j.nut.2016.08.005

[58] Everard, A. et al. Intestinal epithelial MyD88 is a sensor switching host metabolism towards obesity according to nutritional status. *Nat. Commun.* **5**, 5648 (2014). 25476696 10.1038/ncomms6648 PMC4268705

[59] Andrikopoulos, P. et al. Evidence of a causal and modifiable relationship between kidney function and circulating trimethylamine *N*-oxide. *Nat. Commun.* **14**, 5843 (2023). 37730687 10.1038/s41467-023-39824-4 PMC10511707

[60] Hoyles, L. et al. Regulation of blood–brain barrier integrity by microbiome-associated methylamines and cognition by trimethylamine *N*-oxide. *Microbiome* **9**, 235 (2021). 34836554 10.1186/s40168-021-01181-z PMC8626999

[61] Chou, R.-H. et al. Paradox of trimethylamine-*N*-oxide, the impact of malnutrition on microbiota-derived metabolites and septic patients. *J. Intensive Care* **9**, 65 (2021). 34674768 10.1186/s40560-021-00581-5 PMC8529374

[62] Meyer, K. A. et al. Microbiota-dependent metabolite trimethylamine *N*-oxide and coronary artery calcium in the Coronary Artery Risk Development in Young Adults Study (CARDIA). *J. Am. Heart Assoc.* **5**, e003970 (2016). 27792658 10.1161/JAHA.116.003970 PMC5121500

[63] Yao, M.-E., Liao, P.-D., Zhao, X.-J. & Wang, L. Trimethylamine-*N*-oxide has prognostic value in coronary heart disease: a meta-analysis and dose–response analysis. *BMC Cardiovasc. Disord.* **20**, 7 (2020). 31918665 10.1186/s12872-019-01310-5 PMC6953212

[64] Papandreou, C. et al. Plasma trimethylamine-*N*-oxide and related metabolites are associated with type 2 diabetes risk in the Prevención con Dieta Mediterránea (PREDIMED) trial. *Am. J. Clin. Nutr.* **108**, 163–173 (2018). 29982310 10.1093/ajcn/nqy058 PMC6862602

[65] Li, S. et al. Serum trimethylamine-*N*-oxide is associated with incident type 2 diabetes in middle-aged and older adults: a prospective cohort study. *J. Transl. Med.* **20**, 374 (2022). 35982495 10.1186/s12967-022-03581-7 PMC9389664

[66] Svingen, G. F. T. et al. Prospective associations of systemic and urinary choline metabolites with incident type 2 diabetes. *Clin. Chem.* **62**, 755–765 (2016). 26980210 10.1373/clinchem.2015.250761

[67] Roy, S., Yuzefpolskaya, M., Nandakumar, R., Colombo, P. C. & Demmer, R. T. Plasma trimethylamine-*N*-oxide and impaired glucose regulation: results from The Oral Infections, Glucose Intolerance and Insulin Resistance Study (ORIGINS). *PLoS ONE* **15**, e0227482 (2020). 31940332 10.1371/journal.pone.0227482 PMC6961885

[68] Kong, L. et al. Trimethylamine *N*-oxide impairs β-cell function and glucose tolerance. *Nat. Commun.* **15**, 2526 (2024). 38514666 10.1038/s41467-024-46829-0 PMC10957989

[69] Brial, F. et al. Human and preclinical studies of the host–gut microbiome co-metabolite hippurate as a marker and mediator of metabolic health. *Gut* **70**, 2105–2114 (2021). 33975870 10.1136/gutjnl-2020-323314 PMC8515120

[70] Jaworska, K., Bielinska, K., Gawrys-Kopczynska, M. & Ufnal, M. TMA (trimethylamine), but not its oxide TMAO (trimethylamine-oxide), exerts haemodynamic effects: implications for interpretation of cardiovascular actions of gut microbiome. *Cardiovasc. Res.* **115**, 1948–1949 (2019). 31504256 10.1093/cvr/cvz231

[71] Chen, S. et al. Trimethylamine *N*-oxide binds and activates PERK to promote metabolic dysfunction. *Cell Metab.* **30**, 1141–1151.e5 (2019). 31543404 10.1016/j.cmet.2019.08.021

[72] Laakso, M. & Kuusisto, J. Insulin resistance and hyperglycaemia in cardiovascular disease development. *Nat. Rev. Endocrinol.* **10**, 293–302 (2014). 24663222 10.1038/nrendo.2014.29

[73] Ahmetaj-Shala, B. et al. A bioassay system of autologous human endothelial, smooth muscle cells, and leukocytes for use in drug discovery, phenotyping, and tissue engineering. *FASEB J.* **34**, 1745–1754 (2020). 31914612 10.1096/fj.201901379RR PMC6972557

[74] Latorre, J. et al. Decreased lipid metabolism but increased FA biosynthesis are coupled with changes in liver microRNAs in obese subjects with NAFLD. *Int. J. Obes.* **41**, 620–630 (2017). 10.1038/ijo.2017.21 28119530

[75] Toye, A. A. et al. Subtle metabolic and liver gene transcriptional changes underlie diet-induced fatty liver susceptibility in insulin-resistant mice. *Diabetologia* **50**, 1867–1879 (2007). 17618414 10.1007/s00125-007-0738-5

[76] Geurts, L., Muccioli, G. G., Delzenne, N. M. & Cani, P. D. Chronic endocannabinoid system stimulation induces muscle macrophage and lipid accumulation in type 2 diabetic mice independently of metabolic endotoxaemia. *PLoS ONE* **8**, e55963 (2013). 23393605 10.1371/journal.pone.0055963 PMC3564911

[77] Gentleman, R., Carey, V. J., Huber, W., Irizarry, R. A. & Dudoit, S. (eds) *Bioinformatics and Computational Biology Solutions Using R and Bioconductor* (Springer, 2005).

[78] Smyth, G. K. limma: linear models for microarray data. In *Bioinformatics and Computational Biology Solutions Using R and Bioconductor* (eds Gentleman, R. et al.) 397–420 (Springer-Verlag, 2005).

[79] Chen, E. Y. et al. Enrichr: interactive and collaborative HTML5 gene list enrichment analysis tool. *BMC Bioinformatics* **14**, 128 (2013). 23586463 10.1186/1471-2105-14-128 PMC3637064

[80] Tarca, A. L. et al. A novel signaling pathway impact analysis. *Bioinformatics* **25**, 75–82 (2009). 18990722 10.1093/bioinformatics/btn577 PMC2732297

[81] Lee, J.-M. et al. Serum amyloid A3 exacerbates cancer by enhancing the suppressive capacity of myeloid-derived suppressor cells via TLR2-dependent STAT3 activation: cellular immune response. *Eur. J. Immunol.* **44**, 1672–1684 (2014). 24659444 10.1002/eji.201343867

[82] Plovier, H. et al. A purified membrane protein from *Akkermansia muciniphila* or the pasteurized bacterium improves metabolism in obese and diabetic mice. *Nat. Med.* **23**, 107–113 (2017). 27892954 10.1038/nm.4236

[83] Blaise, B. J. et al. Metabotyping of *Caenorhabditis elegans* reveals latent phenotypes. *Proc. Natl Acad. Sci. USA* **104**, 19808–19812 (2007). 18077412 10.1073/pnas.0707393104 PMC2148380

[84] Cheng, Y. & Prusoff, W. H. Relationship between the inhibition constant (K1) and the concentration of inhibitor which causes 50 per cent inhibition (I50) of an enzymatic reaction. *Biochem. Pharmacol.* **22**, 3099–3108 (1973). 4202581 10.1016/0006-2952(73)90196-2

