# Supplementary material for: Inhibition of IRAK4 by microbial trimethylamine blunts metabolic inflammation and ameliorates glycemic control
Source: Nat Metab. 2025 Dec 8;7(12):2531–47. doi: 10.1038/s42255-025-01413-8 (PMC12727497; doi:10.1038/s42255-025-01413-8)
Supplement: Supplementary file 1 — Reporting Summary [file 42255_2025_1413_MOESM1_ESM.pdf]

nature portfolio

Corresponding author(s): Prof. ME Dumas

Last updated by author(s): 2025/09/15

Reporting Summary

Nature Portfolio wishes to improve the reproducibility of the work that we publish. This form provides structure for consistency and transparency in reporting. For further information on Nature Portfolio policies, see our Editorial Policies and the Editorial Policy Checklist.

Statistics

For all statistical analyses, confirm that the following items are present in the figure legend, table legend, main text, or Methods section.

n/a	Confirmed	
☐	☒	The exact sample size (*n*) for each experimental group/condition, given as a discrete number and unit of measurement
☐	☒	A statement on whether measurements were taken from distinct samples or whether the same sample was measured repeatedly
☐	☒	The statistical test(s) used AND whether they are one- or two-sided<br>*Only common tests should be described solely by name; describe more complex techniques in the Methods section.*
☐	☒	A description of all covariates tested
☐	☒	A description of any assumptions or corrections, such as tests of normality and adjustment for multiple comparisons
☐	☒	A full description of the statistical parameters including central tendency (e.g. means) or other basic estimates (e.g. regression coefficient) AND variation (e.g. standard deviation) or associated estimates of uncertainty (e.g. confidence intervals)
☐	☒	For null hypothesis testing, the test statistic (e.g. *F*, *t*, *r*) with confidence intervals, effect sizes, degrees of freedom and *P* value noted<br>*Give P values as exact values whenever suitable.*
☒	☐	For Bayesian analysis, information on the choice of priors and Markov chain Monte Carlo settings
☒	☐	For hierarchical and complex designs, identification of the appropriate level for tests and full reporting of outcomes
☐	☒	Estimates of effect sizes (e.g. Cohen's *d*, Pearson's *r*), indicating how they were calculated

Our web collection on statistics for biologists contains articles on many of the points above.

Software and code

Policy information about availability of computer code

Data collection	MassLynxTM (Waters corporation; Version 4.2) software was used for UPLC-MS/MS data acquisition and analysis. 1H-NMR absolute quantifications were derived using the "In Vitro Diagnostics for research" (IVDr) algorithm (Bruker; v1.1). Image analyses of western blot films and immunohistochemistry slides was conducted with ImageJ (NIH; v1.46r)
Data analysis	Analysis was conducted using the R (v4.03) statistical language as described in the Methods. No custom software was used in this project.

For manuscripts utilizing custom algorithms or software that are central to the research but not yet described in published literature, software must be made available to editors and reviewers. We strongly encourage code deposition in a community repository (e.g. GitHub). See the Nature Portfolio guidelines for submitting code & software for further information.

Data

Policy information about availability of data

All manuscripts must include a data availability statement. This statement should provide the following information, where applicable:

- Accession codes, unique identifiers, or web links for publicly available datasets
- A description of any restrictions on data availability
- For clinical datasets or third party data, please ensure that the statement adheres to our policy

Microarray data are deposited in ArrayExpress under accession number E-MEXP-1755. 1H-NMR mouse metabolomics have been uploaded to Metabolights with

accession number MTBLS12989 (https://www.ebi.ac.uk/metabolights/MTBLS12989). The UPLC-MS/MS spectra for Isotopically quantified methylamines have been deposited to Metabolights with accession number MTBLS12975 (https://www.ebi.ac.uk/metabolights/MTBLS12975).

Research involving human participants, their data, or biological material

Policy information about studies with human participants or human data. See also policy information about sex, gender (identity/presentation), and sexual orientation and race, ethnicity and racism.

Reporting on sex and gender	PBMCs were isolated from 25 - 32 year old female healthy (by self-declaration) volunteers.
Reporting on race, ethnicity, or other socially relevant groupings	Please see below
Population characteristics	Subjects were used only for PBMCs isolation. Since PBMCs from the same participant served as the control (no LPS and/or TMA challenge) and the treatment groups in each independent biological repeat of the experiments in Figure 4 no covariate adjustment was necessary.
Recruitment	Members of the Department of Metabolism Digestion and Reproduction, Imperial (N=4) volunteered to provide blood after a request circulated within the Department. Volunteers received no financial compensation.
Ethics oversight	Imperial College Research Ethics Committee (19IC5372).

Note that full information on the approval of the study protocol must also be provided in the manuscript.

Field-specific reporting

Please select the one below that is the best fit for your research. If you are not sure, read the appropriate sections before making your selection.

☒ Life sciences ☐ Behavioural & social sciences ☐ Ecological, evolutionary & environmental sciences

For a reference copy of the document with all sections, see nature.com/documents/nr-reporting-summary-flat.pdf

Life sciences study design

All studies must disclose on these points even when the disclosure is negative.

Sample size	No sample-size calculation was performed. Sample sizes for the animal experiments were based on previous extensive knowledge of the authors with similar experimental designs to decipher the impact of microbial metabolites on the host (e.g.: please see our study for the metabolite hippurate Brial, F. et al. Gut 70, 2105–2114 (2021)).
Data exclusions	No data where excluded from the animal studies. In the PBMC experiments (Figure 4) one data point was excluded (with high baseline IL6 and TNFa) after the volunteer reported subsequently to the experiment that had received COVID vaccination 3 days prior blood collection.
Replication	To replicate the main finding of our study suggesting a direct inhibitory effect of TMA on IRAK4 kinase activity resulting in alleviated metabolic inflammation and Insulin Resistance (IR) in the host we used multiple distinct but mutually supporting experimental approaches. Specifically, mice were treated with high-choline high-fat diet (HC-HFD which would increase TMA through the microbiome) with or without antibiotics or DMB (an inhibitor of choline conversion to TMA by the microbiome). The beneficial effect of HC-HFD on host glucose handling and IR were abolished by antibiotics or DMB and were emulated by TMA challenge in separate animal experiments. IRAK4 knockout in mice or pharmacological inhibition of its activity by a specific inhibitor also had a beneficial effect on host IR and glucose handling similarly to TMA treament (which would suppress IRAK4 kinase activity). TMA was shown to bind to purified IRAK4 in a pan-kinase screen and to inhibit dose-dependently purified IRAK4 kinase activity. Finally, TMA suppressed cytokine release by human PBMCs stimulated by LPS and rescued an LPS-challenged mouse septic shock model; both processes dependent on IRAK4 kinase activity. Collectively, through a battery of independent experiments ranging from purified proteins to cells and animals we extensively replicated the main thrust of our study i.e: TMA improves host metabolic health by inhibiting IRAK4 kinase activity.<br><br>For the cellular studies there were at least 3 independent biological repeats. All animal studies were conducted once with 6-10 animals per experimental group. All animal experimental groups were treated concurrently.
Randomization	In the animal study, mice were randomly assigned to experimental groups.
Blinding	Where possible experimentalists were blinded. This is clearly stated in the Methods for each relevant experiment.

Reporting for specific materials, systems and methods

We require information from authors about some types of materials, experimental systems and methods used in many studies. Here, indicate whether each material, system or method listed is relevant to your study. If you are not sure if a list item applies to your research, read the appropriate section before selecting a response.

Materials & experimental systems

n/a	Involved in the study
☐	☒ Antibodies
☐	☒ Eukaryotic cell lines
☒	☐ Palaeontology and archaeology
☐	☒ Animals and other organisms
☒	☐ Clinical data
☒	☐ Dual use research of concern
☒	☐ Plants

Methods

n/a	Involved in the study
☒	☐ ChIP-seq
☒	☐ Flow cytometry
☒	☐ MRI-based neuroimaging

Antibodies

Antibodies used	Membranes were immunoblotted with antibodies against the following proteins: pThr345/Ser346IRAK4 (#11927), IRAK4 (#4363), pSer176/180IKKαβ (#2694), IKKβ (#8943), pThr183/Tyr185SAPK/JNK (4668), SAPK/JNK (#9258), pThr180/Tyr182p38MAPK (#9215), p38MAPK (#9212) p-Akt Ser473 (1:1,000; #4060), Total Akt (1:1000 ,#9272S), Total NF- κB p65 (1:3000, #8242), all purchased from Cell Signaling Technology, Inc (CST, MA, USA); β-actin (sc-47778, Santa Cruz Biotechnology, CA, USA) and p-NF-κB (1:3000, #ab86299) from AbCam.
Validation	All the antibodies were sourced commercially and extensively reported in the literature and validated by the vendors. Therefore, no specific validation was performed.

Eukaryotic cell lines

Policy information about cell lines and Sex and Gender in Research

Cell line source(s)	PBMCs were isolated from female volunteers (25-32 years old).<br><br>Primary human hepatocytes were commercially sourced (Innoprot, Bizkaia, Spain)
Authentication	PBMCs responded to LPS challenge in an IRAK4-dependent manner as extensively reported in the literature. Therefore no other specific validation was carried out.<br><br>Primary human hepatocytes were commercially sourced, had the expected morphology and responded to palmitate similarly to previous reports in the literature. We therefore, did not further validate them.
Mycoplasma contamination	PBMCs were isolated from human volunteers and used within one hour of isolation. Therefore, no mycoplasma testing was performed.<br><br>Primary human hepatocytes (HHs) were commercially sourced and tested by the vendor for mycoplasma. Since HHs were used in experiments within 24h of plating no mycoplasma test was performed.
Commonly misidentified lines<br>(See ICLAC register)	Not applicable

Animals and other research organisms

Policy information about studies involving animals; ARRIVE guidelines recommended for reporting animal research, and Sex and Gender in Research

Laboratory animals	Five to six week-old C57BL/6J male mice at the start of the experiment were used in this study.
Wild animals	Not applicable
Reporting on sex	Only male mice were used in our study.
Field-collected samples	Not applicable
Ethics oversight	All experimental procedures involving mice were carried out in accordance with U.K. Home Office, Canadian Council on Animal Care, the ethics committee of the French Research Ministry (authorization number 00486.01), Belgian Law of May 29, 2013 regarding the protection of laboratory animals (agreement number LA1230314) and local guidelines on animal welfare and license conditions and the University of Oxford, University of Ottawa, Université Pierre et Marie Curie and Université catholique de Louvain guidelines on animal welfare. For the septic shock study the animal experiment protocol was approved by local and national committees in charge (Tor Vergata University Institutional Animal Care and Use Committee and Ministry of Health, license no. 265/2019-PR).

Note that full information on the approval of the study protocol must also be provided in the manuscript.

Plants

Seed stocks	Not applicable
Novel plant genotypes	Not applicable
Authentication	Not applicable
